# Uncovering the *Arabidopsis thaliana *nectary transcriptome: investigation of differential gene expression in floral nectariferous tissues

**DOI:** 10.1186/1471-2229-9-92

**Published:** 2009-07-15

**Authors:** Brian W Kram, Wayne W Xu, Clay J Carter

**Affiliations:** 1 Department of Biology, University of Minnesota Duluth, Duluth, MN 55812, USA; 2 Minnesota Supercomputing Institute, University of Minnesota, Minneapolis, MN 55455, USA

## Abstract

**Background:**

Many flowering plants attract pollinators by offering a reward of floral nectar. Remarkably, the molecular events involved in the development of nectaries, the organs that produce nectar, as well as the synthesis and secretion of nectar itself, are poorly understood. Indeed, to date, no genes have been shown to directly affect the *de novo *production or quality of floral nectar. To address this gap in knowledge, the ATH1 Affymetrix^® ^GeneChip array was used to systematically investigate the Arabidopsis nectary transcriptome to identify genes and pathways potentially involved in nectar production.

**Results:**

In this study, we identified a large number of genes differentially expressed between secretory lateral nectaries and non-secretory median nectary tissues, as well as between mature lateral nectaries (post-anthesis) and immature lateral nectaries (pre-anthesis). Expression within nectaries was also compared to thirteen non-nectary reference tissues, from which 270 genes were identified as being significantly upregulated in nectaries. The expression patterns of 14 nectary-enriched genes were also confirmed via RT PCR. Upon looking into functional groups of upregulated genes, pathways involved in gene regulation, carbohydrate metabolism, and lipid metabolism were particularly enriched in nectaries versus reference tissues.

**Conclusion:**

A large number of genes preferentially expressed in nectaries, as well as between nectary types and developmental stages, were identified. Several hypotheses relating to mechanisms of nectar production and regulation thereof are proposed, and provide a starting point for reverse genetics approaches to determine molecular mechanisms underlying nectar synthesis and secretion.

## Background

Nectar is the principal reward offered by flowering plants to attract pollinators [1]; this sugary solution is secreted from floral organs known as nectaries. The complexity of nectar composition has been revealed through many studies on a wide variety of species. In addition to simple sugars (ranging from 8% up to 80%, (w/w) [2]), nearly all nectars contain an assortment of ancillary components, including: amino acids [3], organic acids [4], terpenes [5], alkaloids [6], flavonoids [7], glycosides [8], vitamins [9], phenolics [7], metal ions [10], oils [11], free fatty acids [12], and proteins [13]. Surprisingly, the means by which these compounds arise in nectar are poorly defined. Studies conducted on nectariferous tissue (that constituting the nectary) have traditionally focused on nectar composition, nectary anatomy, and physiological aspects of nectar secretion. Only recently has the goal of identifying the genetic mechanisms regulating nectary development, and nectar production, begun to receive more attention.

**Figure 1:**
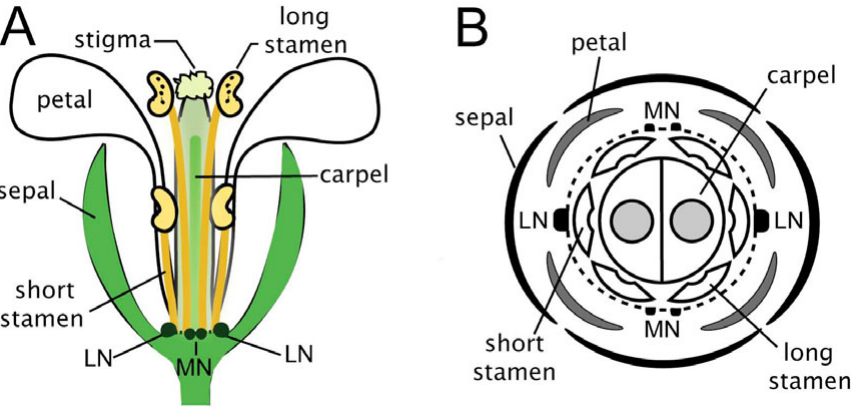
**Schematic of *Arabidopsis thaliana *nectarium**. Arabidopsis flowers have four nectaries that comprise the 'nectarium'; two lateral nectaries (LN) occur at the base of short stamen, and two bilobed median nectaries (MN) occur in between the insertion points of two long stamen. (A) Schematic of Arabidopsis flower with front sepal and petals not shown. (B) Schematic cross-section of flower with relative location of floral organs from (A) indicated (modified from [14]). A narrow ridge of tissue that occasionally connects median and lateral nectaries is indicated with dashed lines. Lateral nectaries produce >95% of total nectar in most Brassicaceae flowers, with median nectaries being relatively non-functional.

The *Arabidopsis thaliana *'nectarium' consists of two pairs of nectaries, lateral and median (see Figure 1; [14]). The two lateral nectaries (LN) are longitudinally opposed to one another just outside the base of short stamen, and are bounded by petal insertion sites. The two median nectaries (MN) also occur on opposite sides of the flower but only between the insertion points of two long stamen. Interestingly, these two nectary types are morphologically and functionally distinct, with lateral nectaries producing the bulk of the nectar (on average >95% of total nectar carbohydrate), and median nectaries producing little or no nectar [14]. While lateral nectaries are regularly supplied with an abundance of phloem, by comparison, the median nectaries are subtended by only a small number of sieve tubes [15].

Despite the near absence of genetic information about the regulation of nectary form and function, some aspects of nectary biology have been extensively studied. For example, the morphology of nectaries from a number of species has been closely examined and, as a result, there is a clear understanding (down to the ultrastructural level) of some of the processes that occur in nectariferous tissue (reviewed in [16]). For example, at the onset of nectar production and secretion in Arabidopsis, small vacuoles, in a dense cytoplasm, are evident in presecretory nectariferous cells [17]. As these cells begin to actively secrete nectar, vacuole size, endoplasmic reticulum activity, and mitochondrial number all increase [17-19]. Conversely, dictyosome number decreases and plastid starch grains, which presumably serve as a source of nectar carbohydrate, also become smaller immediately before secretion [17-20]. In addition, nectary cells likely have high levels of cellular respiration, as evidenced by the abundance of mitochondria with well-developed cristae in nectaries from multiple species [15,21]. While these ultrastructural features of Arabidopsis nectaries are known, the precise physical mechanism of secretion is still an open question [16].

A prevailing view of merocrine-type nectar secretion, used by Arabidopsis and most other nectar producing plants, suggests that some or nearly all pre-nectar metabolites (originating from the phloem sap) are transported symplastically (between cells) via plasmodesmata in nectary parenchyma cells. Here they are stored in secretory cells at or near the nectary surface [21-23]. Immediately prior to secretion, it is thought that starch grains are degraded and most metabolites are packaged into endoplasmic reticulum (ER) and/or Golgi-derived vesicles and secreted via fusion with the plasma membrane (granulocrine secretion). In fact, ultrastructural analyses have repeatedly demonstrated the presence of extensive ER and Golgi networks in nectary secretory cells [16,17,21,22,24]. The model described above does not necessarily discount the direct involvement of plasma membrane transporters in the movement of solutes into nectar (eccrine secretion). Interestingly, a number of plant species, including Arabidopsis, have nectaries with large numbers of modified stomata on their epithelia [25]. It is presumed these stomata are the location where direct nectar secretion from the nectary occurs.

To date, only a few individual genes have been associated with aspects of nectary development: *CRABS CLAW, BLADE-ON-PETIOLE (BOP) 1 *and *BOP2 *[26-29]. *crc *knockout mutants fail to develop nectaries, whereas *bop1/bop2 *double mutant lines have significantly smaller nectaries along with aberrant morphologies [26,29]. While, *CRC *expression alone is necessary, it will not promote ectopic nectary development; this indicates that additional genetic elements might exist that restrict nectary development to the third whorl of the Arabidopsis flower [27]. Other floral organ identity genes have demonstrated or proposed roles in regulating *CRC *expression, although none of these genes alone are required for normal nectary development. Some of these genes include: *LEAFY*, *UFO*, *AGAMOUS*, *SHATTERPROOF1*/*2*, *APETALA2/3*, *PISTILLATA*, and *SEPALLATA1/2/3 *[27,28,30]. In addition to the above, a number of nectary-enriched genes have been identified from multiple species (e.g., [31-39]).

The currently small picture of transcription factors and their downstream targets in nectaries limits our understanding of pathways and cellular processes critical for nectary development and function. Thus, a genome-wide evaluation of gene expression in nectaries could shed some light on key mediators of nectar production. Microarrays have been used to examine gene expression in a wide variety of tissues, and under a broad set of conditions, in Arabidopsis (e.g., [40,41]). However, to date, no genome-wide information on gene expression in nectaries has been reported for Arabidopsis, or any other species. The current lack of global gene expression profiles for nectariferous tissue could possibly be linked to the diminutive nature of Arabidopsis nectaries (at anthesis, lateral nectaries contain roughly 2,000 cells, while median nectaries contain around 400 [27]) and the laborious process associated with manual nectary collection.

Arabidopsis flowers are highly self-fertile, which begs the question as to why these plants would bother to develop functional nectaries; however, solitary bees, flies, and thrips do visit Arabidopsis flowers in the wild, and a small amount of outcrossing does occur [42]. Significantly, many Brassicaceae species (e.g., *Brassica rapa, B. oleraceae*) share similar nectarium structure with Arabidopsis, and produce relatively large amounts of nectar [14,43]. In general, these species are highly dependent on pollinator visitation to achieve efficient pollination [44-47]. Arabidopsis nectaries also appear to share similar developmental mechanisms with a large portion of the eudicot clade [30]. Thus, Arabidopsis, with its fully sequenced genome and genetic resources, can serve as a valuable model for examining nectary development and function in plants.

Here we describe the isolation, amplification, and labeling of transcripts from Arabidopsis nectaries, leading up to an analysis of temporal and spatial gene expression using Affymetrix^® ^Arabidopsis GeneChip ATH1 arrays. We have employed a large-scale analysis of the Arabidopsis nectary transcriptome in order to develop a more complete picture of the genetic programming fundamental to nectar production and secretion. We identify a subset of genes preferentially expressed in nectaries, and distinguish the gene complement upregulated in actively secreting nectaries compared to immature and non-secretory nectaries. Potential genes and pathways involved in nectary development and function are discussed. The resultant data provide a starting-point for reverse genetics approaches to identify specific genes integral to nectar synthesis and secretion.

## Results

### Nectary samples

Floral nectaries are responsible for producing the complex mixture of compounds found in nectar. Surprisingly, a global picture of gene expression in nectaries is currently lacking; however, Arabidopsis nectaries are loosely connected to adjacent floral tissues and can be manually dissected from local non-nectariferous tissues (e.g., Additional file 1). Individual Arabidopsis nectaries are extremely small, thus ~200–300 nectaries were pooled and processed as single biological replicates as indicated in Table 1 (each replicate was isolated from different plants). Specifically, RNA was isolated from immature lateral nectaries (ILN; pre-secretory), mature lateral nectaries (MLN; secretory), and mature median nectaries (MMN, relatively non-secretory). Typical isolations yielded ~300 to 500 ng of total RNA, and were processed for mircroarray hybridizations following a single round of RNA amplification.

**Table 1:** *Arabidopsis thaliana *nectary tissues used for Affymetrix ATH1 microarray analyses

**Floral stage**^a^	**Tissue source**	**Replicates**
14–15 (post-anthesis)	Mature lateral nectary (MLN; secretory)	3
14–15 (post-anthesis)	Mature median nectary (MMN; non-secretory)	2
11–12 (pre-anthesis)	Immature lateral nectary (ILN; pre-secretory)	3

^a ^As defined by Smyth et al., 1990 [67]

Each of the following parameters demonstrated the quality of hybridization and scanning for all nectary samples: signal gradient severity on each chip was under 0.08; outlier area was less than 0.06%; the 3'/5' ratio of housekeeping genes (GAPDH and ubiquitin) were less than 2.5, 'present' call ranges were 40~50%; average intensity ranged from 304 to 618; and all biological replicates consistently had correlations greater than 96%. After quality evaluation, nectary data were then co-normalized with 51 publicly available .cel files representing 13 tissues at multiple developmental stages (see Additional file 2) [41].

Hybridization data were processed with the Expressionist^® ^Analyst module to call gene expression as 'present' or 'absent' in all biological replicates of the nectary tissues examined (quality setting of 0.04 in Expressionist^® ^Analyst software). The number of genes called 'present' in all replicates for each nectary type were: ILN, 11,246; MLN, 9,748; MMN, 11,358. All together, 12,468 genes were confidently expressed in all replicates of one or more nectary tissues, with 9,066 genes being called 'present' (co-expressed) in all nectary experiments. A full list of 'present' genes, along with normalized probe signal values, can be found in Additional file 3.

### Genes preferentially expressed within nectary tissues

**Figure 2:**
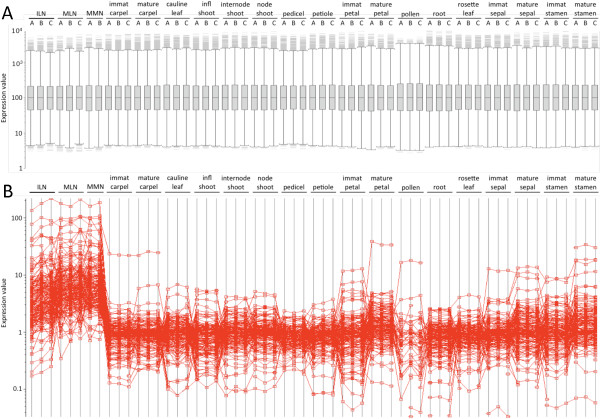
**Signal normalization amongst tissues and resultant clustering**. A box plot representation of signal normalization is presented in panel A. All nectary and non-nectary reference tissue hybridization files (.cel) were quality inspected and then normalized together using the Expressionist^® ^(Genedata, Basel, Switzerland) Refiner module in order to compare gene expression between nectaries and non-nectary tissues. Briefly, .cel files were loaded into Refiner, analyzed and inspected for defective area, average intensity, corner noise, and housekeeping control genes. The probe signals on each .cel file then were quantile normalized and summarized into probe set intensity values by applying the Robust Multiarray Average (RMA) algorithm [69]. Following normalization, signal ratio comparisons between nectaries and reference tissues identified large numbers of genes preferentially expressed within nectaries (panel B), which are presented in Additional file 7.

We foremost wished to identify genes preferentially expressed in nectary tissues since they are likely to be key mediators of nectary development and function. Thus, as mentioned above, we obtained 51 previously published ATH1 array data files representing 13 tissues at multiple developmental stages ([41]; tissues described in Additional file 2). Expression data for all probes were co-normalized to the median probe cell intensity with our nectary samples as described in the *Methods *section (see Figure 2A; full normalized expression data available in Additional file 3). We subsequently calculated normalized signal ratios of individual nectary types against each individual reference tissue. A t-test P value cutoff of 0.05 in probe set signal intensity and a FDR q-value cutoff of 0.1 were initially used to identify genes significantly upregulated in each nectary type over each individual tissue; for downstream analyses, all genes displaying a three-fold or greater increase in probe signal intensity in at least one nectary type (MLN, ILN and/or MMN) over each individual non-nectary reference tissue were determined (the highest observed FDR for any individual 'significant' gene was 0.081; see Table 2 and Additional files 4, 5 and 6). The three-fold cutoff for signal intensity ratio was utilized in this instance to allow a focus on a relatively small number of genes with relatively high enrichment in nectaries, as they are likely key mediators of nectary form and function. A graphical representation of the signal profiles for all 'significant' genes is displayed in Figure 2B. Ultimately, this analysis identified 270 genes upregulated in one or more of the nectary tissues over each individual reference tissue, with the resultant genes being listed in Additional file 7.

**Table 2:** Summary of the identification of nectary-enriched genes

	*Nectary tissues*					
	
	Immature lateral nectary (ILN)		Mature lateral nectary (MLN)		Mature median nectary (MMN)	
*Reference tissues*	Replicates	Significant genes^a^	Replicates	Significant genes^a^	Replicates	Significant genes^a^

Carpel, Immature	3,3	1,053	3,3	2,081	2,3	2,127
Carpel, Mature	3,3	1,059	3,3	2,154	2,3	2,166
Petal, Immature	3,3	714	3,3	1,410	2,3	1,455
Petal, Mature	3,3	1,166	3,3	1,003	2,3	1,061
Sepal, Immature	3,3	1,141	3,3	1,686	2,3	1,697
Sepal, Mature	3,3	1,441	3,3	1,254	2,3	1,291
Stamen, Immature	3,3	1,557	3,3	1,708	2,3	1,720
Stamen, Mature	3,3	1598	3,3	1,120	2,3	1,181
Petiole	3,3	1,157	3,3	2,014	2,3	2,060
Root	3,3	1,826	3,3	2,366	2,3	2,366
Rosette Leaf	3,3	1,268	3,3	1,771	2,3	1,849
Cauline Leaf	3,3	1,319	3,3	1,378	2,3	1,517
Pollen, Mature	3,3	3,923	3,3	3,658	2,3	3,892
Pedicel, Mature	3,3	1,154	3,3	1,918	2,3	2,001
Node Shoot	3,3	1,109	3,3	1,738	2,3	1,760
Internode Shoot	3,3	1,191	3,3	1,358	2,3	1,440
Inflorescence Shoot	3,3	1,271	3,3	2,363	2,3	2,385

Common^b^	---	87	---	198	---	195

^a ^Number of 'present' genes displaying a 3-fold or greater difference in probe signal intensity in ILN, MLN, & MMN over each individual non-nectary reference tissue; a t-test p value cutoff of 0.05, and false discovery rate (FDR) q value cutoff of 0.1 were initially applied to identify genes with significant differences in expression. The highest q value observed for any individual gene after applying the 3-fold cutoff was 0.081.

^b ^The overlapped common gene number represents those genes displaying significant changes that were expressed 3-fold or higher in a given nectary type over all individual reference tissues. The genes identified from this analysis were used to generate Additional file 7; a total of 270 unique genes were found to be upregulated in one or more nectary types over all individual reference tissues.

All plants used for nectary collection were grown under a 16 hour light/8 hour dark cycle, with nectary isolation occurring from 4–8 hours after dawn (h.a.d.). The rationale for this growth and collection scheme was that Arabidopsis flowers fully open by ~3 h.a.d., and nectar production in closely related *Brassica napus *peaks from mid-morning to mid-day (~4 to 8 h.a.d.) [48]. Thus we wished to capture gene expression profiles in nectaries occurring during periods of active secretion. An important item for consideration when evaluating the co-normalized probe signal values described above is that the downloaded AtGenExpress gene expression data (see Additional file 2) were obtained from plants grown under continuous (24 hour) light conditions. Considering that roughly 11% of Arabidopsis genes display diurnal changes in expression (Schaffer et al., 2001), some of the observations in this study may be due to differences in the growth conditions used. Despite the use of different light regimes, comparisons between nectary and AtGenExpress microarray data confirmed the expression of multiple genes known to be upregulated in nectary tissues (see Table 3). Moreover, the expression patterns of multiple nectary-enriched genes identified through comparisons of co-normalized probe signal values were later validated by RT PCR (see below). Finally, there is also precedent in the literature for making this kind of comparison with AtGenExpress data (e.g., [49,50]), which further validates the type of analysis presented here. Thus, while the use of identical growth conditions for all plants would have been ideal for these comparisons, taking advantage of the large publicly available data sets and co-normalizing it with the nectary data presented here provides a means for identifying genes and pathways with nectary-enriched expression profiles.

**Table 3:** Multiple genes with known nectary-enriched expression profiles were confirmed by the microarray experiments

Locus	TAIR annotation	Signal over reference tissue avg.^a^	Signal over next highest tissue^b^	Reference
AT1G19640	S-adenosyl-L-methionine:jasmonic acid carboxyl methyltransferase (JMT)	17.25	2.16 pollen	Song et al., 2000 [37]
AT1G69180	transcription factor CRC (CRABS CLAW)	203.32	30.49 inflor. shoot	Bowman and Smyth, 1999 [26]
AT2G39060	nodulin MtN3 family protein	182.61	39.34 mat. stamen	Ge et al., 2000 [35]
AT2G42830	Agamous-like MADS box protein AGL5 (SHP2)	31.76	6.02 imm. carpel	Savidge et al., 1995 [39]
AT3G25810	terpene synthase/cyclase family protein	302.32	65.27 mat. stamen	Tholl et al., 2005 [38]
AT3G27810	myb family transcription factor (MYB3) (MYB21)	9.39	2.28 mature petal	Jackson et al., 1991 [36]
AT3G58780	Agamous-like MADS box protein AGL1/shatterproof 1 (AGL1) (SHP1)	15.13	3.74 mature carpel	Lee et al., 2005 [28]
AT4G18960	floral homeotic protein AGAMOUS (AG)	13.22	1.72 imm. stamen	Baum et al., 2001 [27]

^a ^Average probe signal in nectaries (MLN, ILN, and MMN combined) over combined average probe signals for all reference tissues described in Additional file 2.

^b ^Full normalized probe signals for all genes called 'present' in nectary tissues are available in Additional file 3. An analysis of all nectary-enriched genes is described in Table 2 and Additional files 4, 5, 6 and 7.

### Differential expression of genes between nectary types and developmental stages

**Figure 3:**
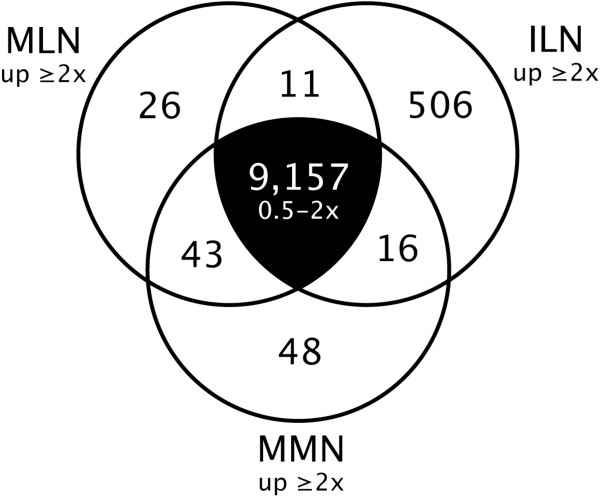
**Comparison of gene expression in different nectary types**. The number of genes displaying a two-fold or greater difference in expression in different nectary types is indicated (e.g., two-fold higher in MLN over ILN and MMN; equal variance two-tailed t-test, p < 0.05; FDR q < 0.05). Genes having similar expression levels in all nectaries types were also determined (0.5 – two-fold difference, center portion of the diagram). An additional 2,661 genes displayed fold changes greater than two between nectary tissues; however, these changes were not statistically significant (p or q > 0.05). Full results are available in Additional file 8, and lists of genes displaying five-fold or greater changes between MLN versus ILN and MMN versus MLN are shown in Additional files 9 & 10, respectively.

Individual nectary types were also compared to one another to identify differentially expressed genes, which may be involved in nectary maturation and nectar secretion. For example, all genes 'present' in at least one nectary type and displaying a two-fold or greater difference in expression between different nectary types were determined (p < 0.05, q < 0.05; see Figure 3 and Additional file 8). Genes having similar expression levels in all nectary types were also identified (0.5 – two-fold difference, 9,157 genes). An additional 2,661 genes displayed fold changes greater than two between nectary tissues; however, these changes were not statistically significant (p or q > 0.05).

For a more in-depth analysis of genes displaying the largest differences in expression, lists of genes displaying five-fold or greater differences in expression level between MLN versus ILN, and MLN versus MMN, are shown in Additional files 9 and 10, respectively. The difference in gene expression between immature lateral nectaries (ILN; pre-secretory) and mature lateral nectaries (MLN; secretory) was substantial, with 335 genes displaying five-fold or greater signal ratios between the two sample types (see Additional file 9). Conversely, the signal profiles of MLN and mature median nectaries (MMN; non-secretory) were remarkably similar, with only 25 genes displaying a five-fold or greater difference (see Additional file 10). Amongst these 25 genes, only a single gene (At2g16720, myb family transcription factor) had at least a five-fold higher signal value in MLN compared to MMN; the remaining 24 differentially expressed genes were five-fold or higher in MMN over MLN. Again, for Additional files 9 &10, genes were manually compiled into ontology groups pertinent to nectary development and function based upon functional analysis, TAIR annotations, and literature searches.

### Validation of gene expression

**Figure 4:**
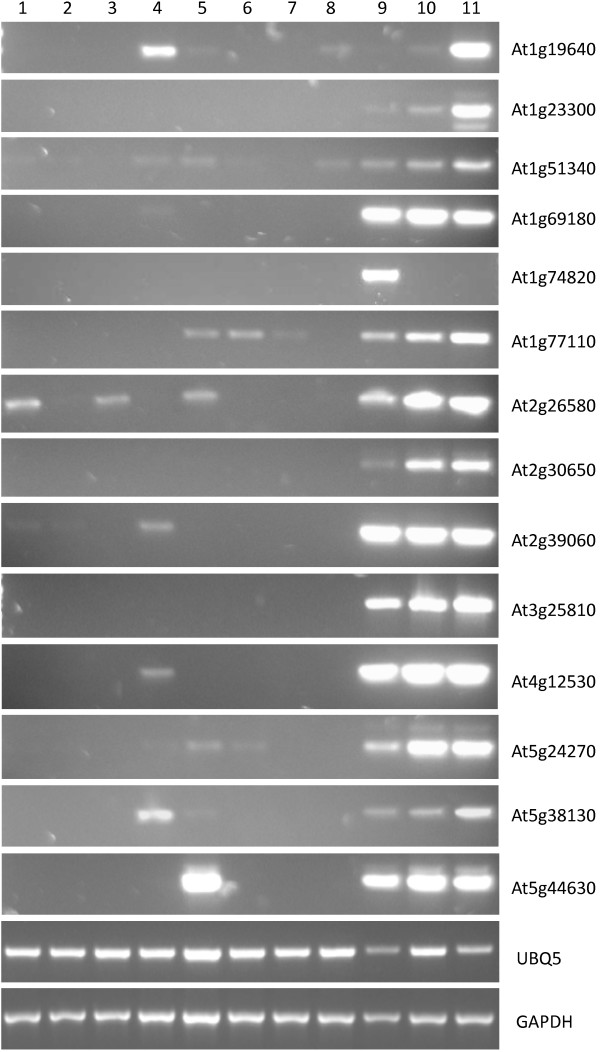
**RT PCR validation of expression profiles**. Reverse transcription-polymerase chain reaction (RT PCR) was used to validate the nectary-enriched expression profiles of select genes identified through microarray analyses. The tissues examined included: 1) petal; 2) sepal; 3) rosette leaf; 4) stamen; 5) pistil; 6) root; 7) internode shoot; 8) silique; 9) mature median nectaries; 10) immature lateral nectaries; and, 11) mature lateral nectaries. Individual genes are described throughout the text and in Additional files 7, 9, and 10; UBQ5 (At3g62250) and GAPDH (At3g04120) were used as constitutively expressed controls.

To validate the expression patterns observed by microarray, RT PCR was utilized. RNA was isolated from 11 tissues (including nectaries) generally represented within our normalized data sets, reverse transcribed, and subjected to PCR. Results shown in Figure 4 demonstrate the nectary-enriched nature of 14 genes, with several of the genes also supporting the changes observed between nectary types via microarray (e.g., At1g19640, At1g74820). Several other pieces of evidence support this overall analysis: 1) promoter::reporter fusions and *in situ *hybridizations previously confirmed the nectary-enriched expression of multiple genes reported here (e.g., [29,38,51,52], Carter et al., *in preparation*); and, 2) an examination of over 11,000 *Brassica rapa *ESTs derived from nectary cDNA libraries, along with corresponding RT PCR analyses, also back the current findings (Hampton et al., *in preparation*).

As another test of the veracity of this type of co-normalization and subsequent analysis, the expression values for eight genes with known nectary-enriched expression profiles were examined (see Table 3). Each of these genes had a minimum nine-fold greater probe signal value in nectaries (ILN, MLN, MMN combined) over the reference tissue average, with individual nectary types displaying higher expression levels over most individual reference tissues (see Additional files 4, 5 and 6).

### Biological processes enriched in nectaries

**Figure 5:**
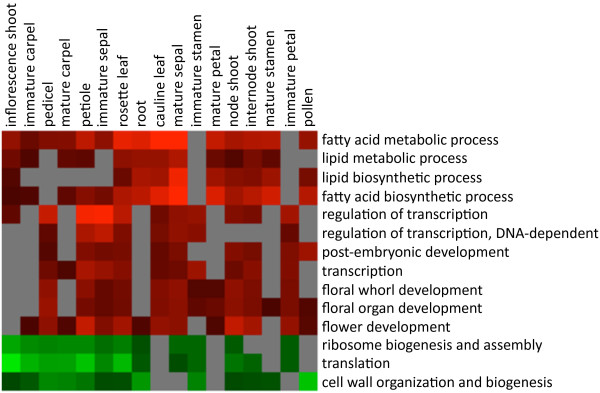
**GO biological process categories significantly enriched or depleted amongst genes upregulated in nectaries**. All genes displaying significant upregulation in all nectary samples (ILN, MLN & MMN) over reference tissues (>3-fold) were placed into GO Biology Process categories via the latest Affymetrix annotation file. Processes showing significant differences between tissues were identified (see Additional file 11) and are graphically represented here. Full graphical results of this analysis are available in Additional file 12.

All genes commonly upregulated in nectaries (MLN, ILN & MMN), when compared to individual reference tissues (>3-fold so as to focus on highly nectary enriched genes), were assigned into GO biological process categories. Processes showing significant differences between tissues were identified (see Additional file 11) and are graphically represented by the heat maps displayed in Figure 5 (condensed) and Additional file 12 (full analysis). This analysis identified several biological processes overrepresented amongst nectary-enriched genes. The biological processes particularly overrepresented in nectaries, when compared to reference tissues, fell within the general categories of lipid and fatty acid biosynthesis and metabolism (see Figure 5). A number of upregulated genes putatively relating to these processes are discussed below.

Transcription processes were also apparently enriched within nectaries (see Figure 5). For example, 45 known and putative transcription factors were found to have enriched expression in one or more nectary types versus non-nectary tissues (see Additional file 7), with a significant subset showing differential expression between nectaries (see Additional files 9 and 10). A number of previous studies have implicated various transcription factors in nectary development, all displaying apparently high expression within nectaries [27,28,30]. Indeed, these findings are reflected in our results, with *CRC *(At1g69180; >200-fold higher in nectaries over reference tissue average), *AGL5*/*SHP2 *(At2g42830, 32-fold), *AGL1/SHP1 *(At3g58780, 15-fold), *AGAMOUS *(At4g18960, 13-fold) and *APETALA2 *(At4g36920, 11-fold) all showing nectary-enriched expression profiles. Curiously, while transcription processes were overrepresented, translation processes were apparently depleted amongst the upregulated genes (see Figure 5).

### The canonical sucrose biosynthesis pathway is upregulated in nectaries

**Figure 6:**
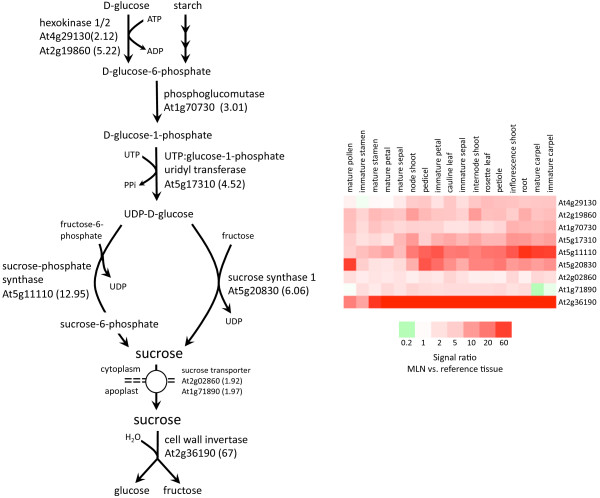
**Genes required for sucrose biosynthesis are upregulated in nectaries**. Genes involved in sucrose biosynthesis, export, and hydrolysis were examined for differential expression between mature lateral nectaries and reference tissues. Individual upregulated genes are labeled within the sucrose biosynthetic pathway (left panel), and the average probe signal value ratio between MLN and reference tissues is shown in parentheses. Most of these genes were significantly upregulated in nectaries over all individual reference tissues, with the heat map (right panel) indicating the relative differences in the probe signal value ratio. The sucrose biosynthetic pathway presented was based on that found in the Plant Metabolic Network (PMN) [74].

Since sugars are the principal solutes in most nectars, it is expected that genes involved in sugar metabolism and transport should be well-represented within the nectary transcriptome. Indeed this is the case, as nearly one dozen sugar metabolizing and modifying genes appear to be preferentially expressed in nectariferous tissues compared to non-nectary tissues (see Additional file 7). In addition, we specifically focused on the expression of genes involved in sucrose metabolism. Results summarized in Figure 6 demonstrate the identification of genes upregulated in nectaries that are putatively involved in sucrose biosynthesis, transport and extracellular hydrolysis. In nearly all instances, these genes had higher probe signal intensities within secretory nectaries (MLN) versus each individual reference tissue (Figure 6 heat map). Experimental evidence has verified the upregulation of both sucrose synthase [51] and cell wall invertase within Arabidopsis nectaries (Ruhlmann et al., *submitted*).

### Identification of promoter motifs within nectary-enriched genes

The large numbers of genes displaying nectary-enriched expression profiles suggests common mechanisms for restricting and/or activating their expression within these secretory organs. An analysis of 96 genes highly and commonly upregulated in multiple nectary types (>10-fold higher probe signal value in ILN, MLN, and/or MMN over the reference tissue average) was performed to identify potential *cis*-acting promoter elements. This analysis identified two DNA sequence motifs particularly overrepresented within the promoters of nectary-enriched genes, MYB4 and CArGCW8GAT. Table 4 displays the relative frequency, location and significance of these elements occurring within the promoters of these genes. Further information on the MYB4 and CArGCW8GAT promoter motifs are discussed below.

**Table 4:** Most common *cis*-acting promoter elements within 96 nectary-enriched genes^a^

Promoter element	Consensus sequence^b^	No. of genes with promoter element	No. of sites within genes	P value
CARGCW8GAT	CWWWWWWWWG			

	From -1 to: -250	28	60	0.0357
	-500	48	122	0.0060
	-750	60	188	0.0020
	-1,000	69	240	10^-3^
	-1,500	82	368	10^-6^
	-2,000	87	466	10^-8^

MYB4	AMCWAMC			

	From -1 to: -250	28	39	0.5974
	-500	51	80	0.1447
	-750	68	120	0.0052
	-1,000	76	151	10^-3^
	-1,500	89	246	10^-7^
	-2,000	94	316	10^-10^

^a ^Analysis performed with *Athena *[76,77]. All overlapping significant genes from Additional file 7 were analyzed [96 genes total displaying 3-fold or greater change in normalized probe signal intensity in two or more nectary types (MLN, MMN, ILN) over all individual reference tissues].

^b ^Where M = A/C and W = A/T

## Discussion

Gene expression profiles in different Arabidopsis tissue types have been extensively compared to one another in order to identify tissue-specific gene expression, especially as it relates to tissue function [40,41]. Significantly, the probe signals from a wide range of independent hybridization experiments can be co-normalized and used to identify differentially expressed genes. For example, the Genevestigator Gene Atlas houses co-normalized probe signal values for ~2,000 Arabidopsis hybridization experiments (from many research groups) representing over 60 different Arabidopsis tissues and cell types [53]. This tool is widely used to examine differential gene expression between tissues, as well as between different growth and treatment conditions, all at the same time (tool currently cited 768 times). However, there is currently no report on gene expression profile comparisons between different nectary tissue types or between nectary and non-nectary tissues.

In this study, we systematically interrogated global differences in gene expression between nectaries and non-nectary reference tissues, as well as between nectary types and developmental stages. Functional classification and analysis of genes upregulated in nectaries versus non-nectary tissues (e.g., Additional file 7), along with genes differentially expressed between secretory and non-secretory nectary tissues (e.g., Additional files 9 and 10) may reveal candidate genes involved in nectar production and secretion. Discussed below are several roles these differentially expressed genes and pathways may play in nectary form and function. To the best of our knowledge, this is the first report of a systematic and global interrogation of any nectary transcriptome.

Not surprisingly, a large number of genes involved in sugar metabolism and processing were differentially expressed between nectary and reference tissues (see Figure 6 and Additional file 7), as well as between nectary tissues themselves (see Additional files 9 and 10). This is in agreement with expectations, as simple sugars are the principal solutes in most nectars. In Arabidopsis phloem sap, the primary sugar is sucrose, while hexoses dominate in the nectar. For example, the sucrose/hexose ratio of Arabidopsis (Col-0) nectar is approximately 0.03 [14]. Resultantly, Arabidopsis nectar would be considered hexose-dominant. The compositional differences between Arabidopsis nectar and phloem photosynthate imply that the phloem "pre-nectar" is modified to yield "mature" nectar, and indeed this proposed process has been supported by a number of studies (as reviewed in [23]). In order to maintain the net flow of carbohydrates from source tissues (e.g. the leaves) to sink tissues like the nectaries, biochemical and physiological processes must be actively maintaining the sink status of nectaries. For example, Bowman [54] noted starch accumulation in Arabidopsis lateral nectaries (Stage 14); specifically, the guard cells showed the most intense staining. Moreover, according to Baum et al. [27], starch-containing plastids are visible in Arabidopsis nectary parenchyma cells from the onset of nectary development, which are apparently degraded just prior to anthesis and nectar secretion [20]. It seems likely that both the modification of phloem sap to nectar and the maintenance of nectaries as a sink tissue are interrelated and even involve many of the same genes.

The coordinated control of sugar transport and metabolism in plant cells and tissues is achieved through the action of sugar modifying enzymes and sugar transporters, both of which play roles in establishing and maintaining sugar concentrations across membranes [55]. For example, invertases are a group of enzymes that hydrolyze sucrose into glucose and fructose, which can then be selectively transported across membranes by hexose transporters and/or help create a sucrose gradient. Significantly, nearly all Arabidopsis invertase genes (both intra- and extracellular) appeared to be upregulated in nectaries, while invertase inhibitor genes seemed to be downregulated in actively secreting nectaries (see Additional file 3). In particular, At2g36190, encoding *Arabidopsis thaliana CELL WALL INVERTASE 4 *(*AtCWINV4*), was strongly upregulated in nectaries (e.g., Figure 6, Additional file 7). Previously, *AtCWINV4 *expression was shown to be high in floral tissues [56]; however, even within floral tissues, expression in nectaries, as observed by microarray, appears pronounced. It is tempting to speculate that this extracellular invertase is at least partly responsible for the hexose-rich nectars observed in Arabidopsis and related members of the Brassicaceae. It may even play a role in maintaining a high intracellular:extracellular sucrose gradient, thus promoting sucrose transport out of nectariferous cells, along with water and other metabolites. Indeed this is likely the case, as *cwinv4 *T-DNA mutants fail to produce nectar and show marked differences in starch accumulation within flowers (Ruhlmann et al., *submitted*).

In addition to invertases, we identified a number of genes upregulated in nectaries involved in other aspects of simple sugar metabolism, with some including: sucrose synthase (*SUS1*, At5g20830; ~5-fold over reference tissues), putative sucrose-phosphate synthase (At5g11110; ~9-fold), putative UDP-glucose 4-epimerase (AT4G23920; ~18-fold), two UDP-glucoronosyl/UDP-glucosyl transferase family proteins (AT5G26310 and AT4G34138; ~14 and 8-fold, respectively) and hexokinase 2 (*HXK2*, AT2G19860; ~4-fold). Significantly, these genes can be tentatively assigned functions in sucrose synthesis/degradation (based upon TAIR AraCyc database, [57]), and are likely involved in defining nectar sugar composition. Indeed, the full canonical sucrose biosynthesis pathway was represented by genes upregulated within mature lateral nectaries over individual reference tissues (see Figure 6). Upregulation of both sucrose synthase [51] and cell wall invertase (Ruhlmann et al., *submitted*) within Arabidopsis nectaries was experimentally verified previously.

Transcription processes were also highly represented within nectary expressed genes (e.g., see Figure 5 and Additional file 11), with 45 of these genes displaying nectary-enriched expression profiles (see Additional file 7). Members of the *YABBY *transcription factor gene family–numbering six in Arabidopsis (*CRABS CLAW*, *FILAMENTOUS FLOWER*, *YABBY3*, *INNER NO OUTER*, *YABBY2*, and *YABBY5*)–are determinants of abaxial cell fate in the lateral floral organs [58]. As previously mentioned, *CRABS CLAW *(At1g69180, *CRC*) encodes a transcription factor involved in the regulation of carpel and nectary development [59]. *CRC *is currently the only known gene to be absolutely required for nectary development; here we have identified several other transcription factors preferentially expressed in nectary tissue that could possibly be involved in either restricting *CRC *expression to the base of the stamens or in some other aspect of nectary development or function. For example, Lee et al. [28] state that there is a "lack of evidence for any other *YABBY *gene family member expressing in the nectaries." However, here we evince the preferential expression of *YABBY5 *(At2g26580) in nectaries, and since this transcription factor belongs to the same family as *CRC*, it too could potentially be involved in mediating nectary development; it had significantly higher signal probe intensities in nectaries over the reference tissue average (~58-fold), and appeared to have relatively constant expression throughout the nectary tissues examined by microarray and RT PCR (see Figure 4).

In addition to transcription factors specifically upregulated in nectaries, some displayed differences between nectary type or developmental stage. For example, the only gene upregulated 5-fold or more in MLN compared to MMN was At2g16720, a myb family transcription factor; probe signal intensity of this gene was also increased greater than 5-fold in MLN over ILN, and 9-fold over the reference tissue average. Since transcription factors modulate the expression of other genes, the involvement of this single gene in differentiating MLN from other tissues could be substantial. Conversely, At4g28140, a putative AP2 domain-containing transcription factor, was upregulated in MMN compared with MLN (8-fold) and ILN (23-fold), and was also upregulated over all reference tissues examined (~20-fold). A separate myb gene (MYB115; At5g40360) was highly expressed in both MLN and MMN, but not ILN, with an overall probe signal increase in nectaries over reference tissues of ~28-fold. Potentially, these genes are involved in differentiating median from lateral, or immature from mature nectaries.

Related to the identification of upregulated transcription factors described above, promoter motifs are short DNA sequences that transcription factors bind to in order to affect the expression of other genes. This is significant within a biological context, as a single transcription factor can simultaneously govern the expression of many other genes (e.g., [60]), provided that the promoter regions of the affected genes contain the DNA sequence motif in question. MYB4 and CArGCW8GAT promoter motifs were particularly overrepresented within the promoters of nectary-enriched genes (see Table 4). Significantly, several CArG boxes were previously identified as key regulators of *CRC *expression within nectaries [28]. The CArG promoter motif (CCWWWWWWGG, where W = A or T) is the canonical target for AGAMOUS and related MADS box proteins, though the CArGCW8GAT motif variant (CWWWWWWWWG) is a known target of AGAMOUS-LIKE MADS BOX PROTEIN 15 (AGL15) specifically. *AGL15 *is primarily expressed in developing embryos [61,62], but is apparently expressed at very low levels within nectaries (data not shown). However, several other MADS box-family genes were highly upregulated in nectaries, including *AGAMOUS *itself, and the functionally redundant *SHATTERPROOF *genes, *AGL1 *and *AGL5 *(see Additional file 7, RT PCR data not shown). These data are consistent with previous findings [27,28,30].

The MYB4 binding motif (AMCWAMC) was also highly represented in the promoters of nectary-enriched genes (316 sites within 94 of 96 promoters analyzed). MYB4 is a direct transcriptional repressor of the cinnamate 4-hydroxylase gene (*C4H*, At2g30490), and can also suppress the expression of chalcone synthase (*CHS*) when overexpressed [63]. C4H and CHS are involved in the synthesis of hydroxycinnamate esters and flavonoids, respectively, both of which are ultimately known to provide protection from UV-B radiation [63,64]. Curiously, by microarray *MYB4 *was highly upregulated within nectaries (see Additional file 7), whereas *C4H *and *CHS *were strongly repressed (by a range of 5 to 100-fold) when compared to reference tissues (see Additional file 3), which supports the known functions of MYB4. Nonetheless, it is tempting to speculate that MYB4, or one of the four other myb family proteins upregulated in nectaries (see Additional file 7), may be involved in the regulation or even activation of nectary-specific genes. Indeed, myb family transcription factors were previously implicated in the regulation of the nectary-specific *NECTARIN 1 *gene in tobacco [32]. While more work needs to be done, the prevalence of MYB4 and CArGCW8GAT promoter motifs within nectary-specific genes suggests that they may provide a basis for regulating nectary-specific gene expression.

Finally, it should be noted that multiple genes involved in aspects of lipid metabolism [e.g., *LTP1 *(At2g38540) and *GPAT5 *(At3g11430)], and auxin transport and response [e.g., *PIN6 *(At1g77110) and *CHY1 *(At2g30650)], were identified as being highly upregulated in nectaries by both microarray and RT PCR. These findings are significant in that both lipid and auxin processes have been suggested to play roles in nectary development and nectar secretion (e.g., [52,65,66]); however, the exact functions these upregulated genes in nectary function is currently unclear.

## Conclusion

By microarray analysis we have identified a large number of genes preferentially expressed in, and between, nectaries. This information now allows for a rapid and targeted reverse genetics approach for identifying key mediators of nectary form and function. Due to its central role in pollination, determining the molecular basis of nectar production can have broad implications, ranging from understanding the co-evolution of plants and animals, to increasing yields in multiple pollinator-dependent crop species.

## Methods

### Plant material and growth conditions

*Arabidopsis thaliana *ecotype Columbia-0 plants were used for this study. Plants were grown in individual pots on a peat-based growth medium with vermiculite and perlite (Pro-Mix BX; Premier Horticulture, Rivière-du-Loup, Quebec, Canada). All plant growth was performed in Percival AR66LX environmental chambers with settings of: 16 hr day/8 hr night cycle, photosynthetic photon flux of 150 μmol m^-2 ^s^-1^, 50% humidity, and temperature of 21°C.

### Nectary sample preparations and RNA isolation

Three different types of RNA samples were prepared from Arabidopsis nectaries: mature lateral nectaries (MLN; Stage 14–15 flowers), immature lateral nectaries (ILN, Stage 11–12 flowers), and mature median nectaries (MMN, Stage 14–15 flowers) (developmental stages defined by Smyth et al. [67]). MLN are secretory tissues, whereas, ILN and MMN are pre-secretory and nonsecretory tissues, respectively. All nectary tissues were separately dissected by hand from the flowers of primary inflorescences of ca. 30–35 day-old plants. Due to the small size of nectaries, dissections took place over several days from 4–8 hours after dawn (h.a.d.). Isolated nectaries were pooled in RNAlater™ solution (Ambion, Austin, TX) on ice, and stored at 4°C prior to RNA extraction. Up to two nectaries were collected per flower, with approximately 200–300 nectaries being processed as a single RNA sample. Each biological replicate was represented by nectaries pooled from different sets of plants. An example of nectary dissection can be viewed in Additional file 1.

### RNA extraction, target synthesis, and hybridization to Affymetrix^® ^GeneChips

RNA was extracted from floral nectariferous tissue by mechanical disruption, with a microcentrifuge pestle, and using the RNAqueous^®^-Micro micro scale RNA isolation kit (Ambion, Austin, TX) with Plant RNA Isolation Aid (Ambion, Austin, TX). Denaturing agarose gel electrophoresis [68] and UV spectrophotometry were used to assess RNA quality for all samples.

RNA was processed for use on Affymetrix^® ^GeneChip Arabidopsis ATH1 genome arrays (Affymetrix, Santa Clara, CA) using MessageAmp™ II-Biotin *Enhanced *Kit (Ambion, Austin, TX) for a single round of RNA amplification as described by the manufacturer. Briefly, 250–500 ng of total RNA (500 ng from lateral nectaries; 250 ng from median nectaries due to extremely small size) was used in a reverse transcription reaction to generate first-strand cDNA. Following second-strand synthesis, double-stranded cDNA was used in an *in vitro *transcription (IVT) reaction to generate biotin-labeled, amplified RNA (aRNA). aRNA size distribution was evaluated by conventional denaturing agarose gel analysis according to manufacturer's instructions (Ambion, Austin, TX). An aRNA fragmentation reaction, employing metal-induced hydrolysis, was used to fragment aRNA as described by the manufacturer (Ambion, Austin, TX). Success of the fragmentation reaction was evaluated via denaturing agarose gel electrophoresis, as indicated above. Fifteen micrograms of fragmented aRNA for each sample was submitted, on dry ice, to the University of Minnesota BMGC Microarray Facility in Minneapolis, Minnesota. Array hybridization and scanning, using a GeneChip 3000 scanner, were performed at the facility.

### Data quality and normalization

Following hybridization, data quality was ensured by examining the 3'/5' ratio of housekeeping genes, the signal intensities and outliers, and the overall 'present' calls of probe sets by using the Expressionist^® ^(Genedata, Basel, Switzerland) Refiner module. The probe signal levels were quantile-normalized and then summarized using the RMA algorithm [69]. Gene expression values were further linearly scaled up to a media of 100 in the Expressionist^® ^(Genedata, Basel, Switzerland) Analyst module. All pertinent data files were submitted to the National Center for Biotechnology Information Gene Expression Omnibus (NCBI GEO).

### Experimental design and statistical analyses

As mentioned above, we used the ATH1 oligonucleotide array to specifically assess gene expression in: 1) immature lateral nectaries (ILN; pre-secretory nectaries from Stage 11–12 pre-anthesis flowers); 2) mature lateral nectaries (MLN; secretory nectaries from Stage 14–15 post-anthesis flowers); and, 3) mature median nectaries (MMN; non-secretory nectaries from Stage 14–15 post-anthesis flowers). This analysis was performed in order to identify genes tentatively involved in nectar production and secretion. Furthermore, we aimed to implicate additional genes in the regulation of nectary development.

Three types of group comparisons were performed in this study: MLN versus ILN to identify developmentally and temporally regulated genes involved in nectar production; MLN versus MMN to identify specific genes potentially involved in nectar production; and MLN, ILN, and MMN versus non-nectary reference tissues to identify nectary-enriched genes. For all analyses we used data generated from pooled nectaries (see above), which was due to the insufficient amount of material available from individual nectaries. Each sample pool contained 200–300 nectaries, with two (MMN) or three (MLN and ILN) biological replicates being performed for each nectary tissue (see Table 1; each replicate was isolated from different plants). T-tests for pooling of samples were applied in these comparisons [70,71]. We justified the false discovery rate (FDR) of the resultant significant gene lists according to Storey and Tibshirani [72].

To identify genes that are specifically upregulated in nectary tissues, and therefore may contribute to nectar production, we compared individual nectary samples (ILN, MLN & MMN) with 13 non-nectary reference tissue data sets (each in triplicate, see Additional file 1). A Welch modified t-test was applied for this unequal variances comparison.

### RT PCR validation

In addition to nectaries, total RNA was extracted from multiple reference tissues for the validation of expression patterns observed by microarray; RNA from all non-nectary floral tissues were dissected from Stage 14–15 flowers. Tissues were collected in RNAlater™ (Ambion, Austin, TX) and stored at 4°C prior to extraction. RNA isolation was performed by mechanical disruption, with a microcentrifuge pestle, and using the RNAqueous^®^-Micro micro scale RNA isolation kit (Ambion, Austin, TX), along with Plant RNA Isolation Aid (Ambion, Austin, TX); the optional DNase I treatment was performed according to the manufacturer's instructions. Standard agarose gel electrophoresis and UV spectrophotometry were used to assess RNA quality for all samples. RNA was reverse transcribed (0.1 μg per tissue) with Promega's (Madison, WI, USA) Reverse Transcription System (A3500), and PCR was performed with GoTaq Green Master Mix (Promega, M7122). Negative control reactions using RNA, without reverse transcription, as template for PCR was used to verify the absence of contaminating genomic DNA in all samples. All primers used in this study are listed in Additional file 13.

### Functional group overrepresentation analysis

In order to examine the known functions and relationships of the differentially expressed genes, we input these genes into Pathway Studio 5.0^® ^(Ariadne Genomics, Rockville, MD) for gene ontology, canonical pathways, and interaction network analysis. Highly expressed tissue-specific genes were mapped to GO Slim (an overall view of gene ontology groups) in order to compare the tissue-specific enriched GO groups. Functional groups pertinent to nectary development and nectar production were then manually inspected and grouped based upon TAIR annotations [73] and literature searches. Evaluation of gene expression in the canonical sucrose biosynthesis pathway (see Figure 6) was performed via the OMICS Viewer of the Plant Metabolic Network (PMN) [74].

Genes commonly upregulated in nectaries (MLN, ILN & MMN; eight samples) versus reference tissues were also identified (3-fold upregulated, Welch's T test P 0.05) and assigned into GO biological process categories (gene ontologies from newest Affymetrix annotation file (ATH1-121501 Annotations; 3/12/09). Fisher's Exact Test in Expressionist software (GeneData) was used determine the significance of nectary-upregulated genes, seemingly overrepresented in a particular GO category when compared against all genes contained in said GO category. In each case Fisher's test indicated whether it was possible to reject the null hypothesis that observed differences are due to chance. We plotted the log transformed Fisher's Test P values onto a heat map using Treeview software [75].

### Promoter motif analysis

To identify *cis*-acting promoter elements potentially involved in regulating the co-expression of genes within nectaries, the *Arabidopsis thaliana expression network analysis *(Athena)tool was used [76,77]. Specifically, the promoter regions of 96 genes displaying significant enrichment in multiple nectary samples were analyzed (i.e., genes from Additional file 7 with >10-fold higher probe signal value in at least two of the three nectary samples). The -2,000 to -1 regions of all promoters were examined, as the expression of *CRC*, a nearly nectary-specific gene, is controlled by elements as distal as -2.5 kb [28].

## List of abbreviations used

MLN: mature lateral nectary; ILN: immature lateral nectary; MMN: mature median nectary.

## Authors' contributions

BWK and CJC designed the experiments. Nectary collection, RNA isolation, RNA processing and RT PCR were carried out by BWK. The majority of bioinformatics and statistical analyses were performed by WWX, with significant contributions from BWK and CJC. The manuscript was written by BWK, WWX and CJC. All authors read and approved the final manuscript.

## Supplementary Material

Additional file 1 **Movie demonstrating nectary isolation**. Example of lateral nectary dissection from Arabidopsis flower. Click here for file

Additional file 2 **Non-nectary reference tissues**. Description of non-nectary reference tissue .cel files used for analyses. Click here for file

Additional file 3 **All normalized probe values**. Full list of normalized probe signal intensities for all genes called 'present' in nectaries (includes values for reference tissues). Click here for file

Additional file 4 **ILN versus reference tissues**. List comparing gene expression between ILN and reference tissues (includes p and q values). Click here for file

Additional file 5 **MLN versus reference tissues**. List comparing gene expression between MLN and reference tissues (includes p and q values). Click here for file

Additional file 6 **MMN versus reference tissues**. List comparing gene expression between MMN and reference tissues (includes p and q values). Click here for file

Additional file 7 **Genes displaying nectary-enriched expression profiles**. All genes displaying a 3-fold or greater change in normalized probe signal intensity in one or more nectary types (MLN, MMN, ILN) over all individual reference tissues are displayed (t-test p-value cutoff 0.05 and FDR q-value cutoff 0.1; data summarized in Table 2). Click here for file

Additional file 8 **Nectary versus nectary comparison**. Filterable list comparing gene expression between different nectary samples. Click here for file

Additional file 9 **Genes displaying differential expression between mature and immature lateral nectaries**. All genes displaying a 5-fold or greater difference in probe signal value between MLN and ILN are shown (t test p-value cutoff 0.05, and FDR q-value cutoff 0.05). Click here for file

Additional file 10 **Genes displaying differential expression between mature median and mature lateral nectaries**. All genes displaying a 5-fold difference in probe signal value between MMN and MLN are shown (t test p-value cutoff 0.05, and FDR q-value cutoff 0.05). Click here for file

Additional file 11 **Gene ontologies for nectary enriched genes**. Full gene ontology analysis for nectary-enriched genes. Click here for file

Additional file 12 **Full gene ontology heat map**. Full heat map of gene ontology analysis for nectary-enriched genes. Click here for file

Additional file 13 **Oligonucleotides used in this study**. List of primers used for RT PCR validation experiments. Click here for file
