# Supplementary material for: Uncovering the Arabidopsis thaliana nectary transcriptome: investigation of differential gene expression in floral nectariferous tissues
Source: BMC Plant Biol. 2009 Jul 15;9:92. doi: 10.1186/1471-2229-9-92 (PMC2720969; doi:10.1186/1471-2229-9-92)
Supplement: Additional file 7 — Genes displaying nectary-enriched expression profiles. All genes displaying a 3-fold or greater change in normalized probe signal intensity in one or more nectary types (MLN, MMN, ILN) over all individual reference tissues are displayed (t-test p-value cutoff 0.05 and FDR q-value cutoff 0.1; data summarized in Table 2). [file 1471-2229-9-92-S7.doc]

| **Additional file 7 - Genes displaying nectary-enriched expression profiles** | | | | | |
| --- | --- | --- | --- | --- | --- |
|  | | | **Fold change over reference tissue avg**^a^ | | |
| **Locus** | **TAIR Annotation** | **Probe Set** | **ILN** | **MLN** | **MMN** |
| *Transcription Factors* | |  |  |  |  |
| AT1G03770 | zinc finger (C3HC4-type RING finger) family protein | 265086_at | 20.68 | 33.56 | 30.30 |
| AT1G07050 | CONSTANS-like protein-related | 256060_at | 9.89 | 3.43* | 4.81* |
| AT1G08000 /// AT1G08010 | zinc finger (GATA type) family protein | 260680_s_at | 2.53* | 5.22 | 5.05 |
| AT1G23380 | homeobox transcription factor (KNAT6) | 263012_at | 2.63* | 9.19* | 15.88 |
| AT1G29220 | transcriptional regulator family protein | 260894_at | 2.13* | 4.89 | 6.01 |
| AT1G32540 | zinc finger protein, putative | 256469_at | 11.99 | 19.37 | 13.07 |
| AT1G62360 | homeobox protein SHOOT MERISTEMLESS (STM) | 260632_at | 9.56* | 16.03 | 12.56* |
| AT1G64620 | Dof-type zinc finger domain-containing protein | 261950_at | 3.49* | 5.88* | 9.71 |
| AT1G69180 | transcription factor CRC (CRABS CLAW) | 260355_at | 176.32 | 198.09 | 251.69 |
| AT1G77850 | transcriptional factor B3 family protein | 262136_at | 7.90 | 11.59 | 13.50 |
| AT1G79700 | ovule development protein, putative | 261395_at | 19.87 | 27.13 | 12.17* |
| AT2G14210 | MADS-box protein (ANR1) | 263295_at | 6.60* | 38.29 | 28.25 |
| AT2G21320 | zinc finger (B-box type) family protein | 263739_at | 1.33* | 10.30 | 10.93 |
| AT2G22680 | zinc finger (C3HC4-type RING finger) family protein | 265345_at | 37.20 | 53.13 | 49.57 |
| AT2G22800 | homeobox-leucine zipper protein 9 (HAT9) / HD-ZIP protein 9 | 266824_at | 9.86* | 31.60 | 26.07 |
| AT2G22850 | bZIP transcription factor family protein | 266798_at | 1.57* | 11.20 | 5.47* |
| AT2G26580 | plant-specific transcription factor YABBY family protein | 245029_at | 43.81 | 68.71 | 64.28 |
| AT2G41900 | zinc finger (CCCH-type) family protein | 267534_at | 2.73* | 5.65 | 5.30* |
| AT2G42660 | myb family transcription factor | 263977_at | 9.79 | 4.84* | 3.35* |
| AT2G42830 | agamous-like MADS box protein AGL5 / floral homeodomain transcription factor (AGL5) | 263988_at | 17.22 | 42.37 | 37.65 |
| AT2G45120 | zinc finger (C2H2 type) family protein | 266133_at | 3.07* | 8.63 | 3.81* |
| AT2G46590 | Dof zinc finger protein DAG2 / Dof affecting germination 2 (DAG2) | 265448_at | 1.64* | 2.65* | 6.55 |
| AT2G46870 | DNA-binding protein, putative | 266760_at | 0.42* | 0.49* | 9.05 |
| AT2G47810 | histone-like transcription factor (CBF/NF-Y) family protein | 257371_at | 14.08 | 37.12 | 30.82 |
| AT3G05200 | zinc finger (C3HC4-type RING finger) family protein (ATL6) | 259312_at | 3.25* | 10.47 | 10.30 |
| AT3G06330 | zinc finger (C3HC4-type RING finger) family protein | 258882_at | 1.60* | 9.82 | 8.39 |
| AT3G09600 | myb family transcription factor | 258724_at | 2.29* | 14.52 | 8.27 |
| AT3G13540 | myb family transcription factor | 256985_at | 4.46* | 5.68* | 8.00 |
| AT3G21270 | Dof-type zinc finger domain-containing protein (ADOF2) | 258044_at | 7.59* | 18.59 | 11.84 |
| AT3G49930 | zinc finger (C2H2 type) family protein | 252236_at | 11.51 | 11.31 | 3.16* |
| AT3G50410 | Dof-type zinc finger domain-containing protein | 252210_at | 0.89* | 10.28 | 4.53* |
| AT3G61460 | zinc finger (C3HC4-type RING finger) family protein (BRH1) | 251321_at | 3.01* | 7.66 | 6.68* |
| AT4G02075 | zinc finger (C3HC4-type RING finger) family protein | 255511_at | 10.63 | 16.45 | 8.12 |
| AT4G08350 /// AT4G08360 | KOW domain-containing transcription factor family protein | 255136_s_at | 2.49* | 4.31 | 3.92 |
| AT4G13100 | zinc finger (C3HC4-type RING finger) family protein | 254803_at | 4.45* | 15.83 | 11.98 |
| AT4G34680 | GATA transcription factor 3, putative (GATA-3) | 253249_at | 20.89 | 32.83 | 27.03 |
| AT4G35480 | zinc finger (C3HC4-type RING finger) family protein | 253140_at | 23.18 | 15.47 | 21.01 |
| AT4G36920 | floral homeotic protein APETALA2 (AP2) | 246217_at | 5.80* | 14.90 | 15.13 |
| AT4G37790 | homeobox-leucine zipper protein 22 (HAT22) / HD-ZIP protein 22 | 253038_at | 8.50 | 10.03 | 9.93 |
| AT4G38620 | myb family transcription factor (MYB4) | 252958_at | 5.69* | 7.82 | 7.97 |
| AT4G38960 | zinc finger (B-box type) family protein | 252917_at | 1.34* | 10.65 | 9.17 |
| AT5G03180 | zinc finger (C3HC4-type RING finger) family protein | 250938_at | 8.16 | 6.84 | 5.05* |
| AT5G40360 | myb family transcription factor (MYB115) | 249350_at | 0.93* | 37.93 | 52.99 |
| AT5G52830 | WRKY family transcription factor | 248306_at | 2.60* | 16.98 | 11.19 |
| AT5G58620 | zinc finger (CCCH-type) family protein | 247795_at | 3.39* | 6.60* | 10.40 |
|  |  |  |  |  |  |
| *Carbohydrate Metabolism* | |  | **ILN** | **MLN** | **MMN** |
| AT2G19860 | hexokinase 2 (HXK2) | 266702_at | 3.30* | 4.33* | 5.22 |
| AT2G36190 | beta-fructosidase, putative / beta-fructofuranosidase, putative | 263905_at | 65.77 | 66.63 | 62.00 |
| AT2G43670 | glycosyl hydrolase family protein 17 | 260611_at | 2.57* | 2.90* | 11.59 |
| AT2G44480 | glycosyl hydrolase family 1 protein | 267391_at | 1.06* | 50.02 | 18.52* |
| AT3G14570 | glycosyl transferase family 48 protein | 258122_at | 5.96 | 6.71 | 2.94* |
| AT3G17130 | invertase/pectin methylesterase inhibitor family protein | 257876_at | 0.34* | 0.89* | 14.01 |
| AT3G26380 | glycosyl hydrolase family protein 27 / alpha-galactosidase family protein / melibiase family protein | 256866_at | 4.52 | 2.55* | 2.10* |
| AT4G23920 | UDP-glucose 4-epimerase, putative / UDP-galactose 4-epimerase, putative / Galactowaldenase, putative | 254188_at | 5.47* | 23.04 | 29.13 |
| AT4G34138 | UDP-glucoronosyl/UDP-glucosyl transferase family protein | 253281_at | 4.40* | 11.29 | 10.42 |
| AT5G11110 | sucrose-phosphate synthase, putative | 245904_at | 2.91* | 11.50 | 12.95 |
| AT5G26310 | UDP-glucoronosyl/UDP-glucosyl transferase family protein | 246826_at | 7.30* | 24.85 | 7.17* |
|  |  |  |  |  |  |
| *Lipid Metabolism & Processing* | |  | **ILN** | **MLN** | **MMN** |
| AT1G33270 | patatin-related | 256534_at | 5.02* | 16.92 | 19.14 |
| AT1G74960 | 3-ketoacyl-ACP synthase, putative | 262176_at | 4.26* | 7.49 | 5.51 |
| AT2G30650 | 3-hydroxyisobutyryl-coenzyme A hydrolase, putative / CoA-thioester hydrolase, putative | 267571_at | 234.44 | 381.26 | 274.91 |
| AT2G36290 | hydrolase, alpha/beta fold family protein | 263900_at | 3.39* | 8.39 | 7.05* |
| AT3G29770 | hydrolase, alpha/beta fold family protein | 256564_at | 6.35 | 6.35* | 3.55* |
| AT4G12530 | protease inhibitor/seed storage/lipid transfer protein (LTP) family protein | 254829_at | 316.88 | 376.33 | 352.76 |
| AT4G14430 | enoyl-CoA hydratase/isomerase family protein | 245359_at | 7.86 | 5.59* | 5.08* |
| AT5G22500 | acyl CoA reductase, putative / male-sterility protein, putative | 249895_at | 16.63 | 12.81 | 10.81 |
| AT5G48880 | acetyl-CoA C-acyltransferase 1 / 3-ketoacyl-CoA thiolase 1 (PKT1) | 248625_at | 7.94* | 11.22 | 9.88* |
| AT5G65110 | acyl-CoA oxidase (ACX2) | 247176_at | 8.19 | 10.03 | 10.25 |
|  |  |  |  |  |  |
| *Transporter and Channel Proteins* | |  | **ILN** | **MLN** | **MMN** |
| AT1G05300 | metal transporter, putative (ZIP5) | 264574_at | 10.39 | 10.17 | 6.98 |
| AT1G15170 | MATE efflux family protein | 262605_at | 4.29* | 14.31 | 9.02* |
| AT1G23300 | MATE efflux family protein | 263040_at | 38.01 | 171.12 | 58.88 |
| AT1G51340 | MATE efflux family protein | 265151_at | 6.23* | 37.65 | 32.61 |
| AT1G69850 | nitrate transporter (NTL1) | 260414_at | 7.24* | 12.87 | 10.77 |
| AT2G01980 | sodium proton exchanger, putative (NHX7) (SOS1) | 265252_at | 11.77 | 16.20 | 9.03 |
| AT2G13650 | GDP-mannose transporter (GONST1) | 263722_at | 2.69* | 9.54 | 7.00 |
| AT2G40540 | potassium transporter, putative (KT2) | 255829_at | 3.21* | 8.29* | 12.70 |
| AT3G53720 | cation/hydrogen exchanger, putative (CHX20) | 251906_at | 15.17* | 16.27 | 8.51* |
| AT3G54820 | aquaporin, putative | 251858_at | 2.21* | 9.70* | 24.10 |
| AT3G62150 | multidrug resistant (MDR) ABC transporter, putative | 251248_at | 1.87* | 12.66 | 8.74 |
| AT4G30560 | cyclic nucleotide-regulated ion channel, putative | 253622_at | 21.56 | 7.80 | 13.61 |
| AT5G14940 | proton-dependent oligopeptide transport (POT) family protein | 246566_at | 0.43* | 2.71* | 23.04 |
| AT5G46540 | ABC transporter family protein | 248849_at | 25.06 | 17.50 | 8.96* |
|  |  |  |  |  |  |
| *Signaling and Development* | |  | **ILN** | **MLN** | **MMN** |
| AT1G21920 | MORN (Membrane Occupation and Recognition Nexus) repeat-containing protein /phosphatidylinositol-4-phosphate 5-kinase-related | 260855_at | 2.46* | 8.44 | 6.44* |
| AT1G55200 | protein kinase family protein | 259656_at | 11.35* | 19.64 | 14.79* |
| AT1G55610 | protein kinase family protein | 264537_at | 16.11 | 10.40* | 21.16 |
| AT1G68795 | CLE12 | 260039_at | 15.82 | 17.27 | 13.77 |
| AT1G69970 | CLE26, putative | 264693_at | 23.79 | 20.11 | 12.32 |
| AT1G71010 | phosphatidylinositol-4-phosphate 5-kinase family protein | 262308_at | 2.07* | 6.01 | 5.21 |
| AT1G71710 | inositol polyphosphate 5-phosphatase, putative | 261522_at | 5.85* | 10.95 | 12.51 |
| AT1G77660 | MORN (Membrane Occupation and Recognition Nexus) repeat-containing protein /phosphatidylinositol-4-phosphate 5-kinase-related | 259730_at | 6.85* | 7.07 | 6.35* |
| AT2G25180 | two-component responsive regulator family protein / response regulator family protein | 264374_at | 2.47* | 3.73* | 4.81 |
| AT2G39920 | acid phosphatase class B family protein | 267361_at | 9.80 | 7.66* | 7.23* |
| AT2G41140 | calcium-dependent protein kinase, putative / CDPK, putative | 267082_at | 7.57 | 8.19 | 9.32 |
| AT3G05640 | protein phosphatase 2C, putative / PP2C, putative | 258901_at | 2.80* | 19.17 | 40.84 |
| AT3G07980 /// AT3G13530 | protein kinase, putative | 258632_s_at | 6.75 | 8.18 | 8.73 |
| AT3G22420 | protein kinase family protein | 258456_at | 10.38 | 10.69 | 9.73 |
| AT3G45780 | protein kinase / nonphototropic hypocotyl protein 1 (NPH1) / phototropin | 252543_at | 8.24 | 11.08 | 10.16 |
| AT3G52290 | calmodulin-binding family protein | 256671_at | 2.94* | 13.08 | 8.89 |
| AT4G30520 | leucine-rich repeat family protein / protein kinase family protein | 253620_at | 16.03 | 5.89* | 1.94* |
| AT4G38810 | calcium-binding EF hand family protein | 252915_at | 0.53* | 9.93 | 19.47 |
| AT5G07070 | CBL-interacting protein kinase 2 (CIPK2) | 250673_at | 0.86* | 2.38* | 4.83 |
| AT5G48640 | cyclin family protein | 248659_at | 5.74* | 7.67 | 5.49* |
| AT5G50120 | transducin family protein / WD-40 repeat family protein | 248535_at | 3.58* | 20.49 | 14.62 |
| AT5G53320 | leucine-rich repeat transmembrane protein kinase, putative | 248254_at | 8.02* | 15.44 | 19.33 |
| AT5G54200 | WD-40 repeat family protein | 248198_at | 3.18* | 7.24 | 4.63* |
| AT5G62070 | calmodulin-binding family protein | 247464_at | 1.49* | 4.37* | 8.51 |
|  |  |  |  |  |  |
| *Hormone Metabolism and Response* | |  | **ILN** | **MLN** | **MMN** |
| AT1G02400 | gibberellin 2-oxidase, putative / GA2-oxidase, putative | 259445_at | 0.30* | 6.09* | 11.38 |
| AT1G19640 | S-adenosyl-L-methionine:jasmonic acid carboxyl methyltransferase (JMT) | 261150_at | 8.12* | 33.04 | 7.24* |
| AT1G29500 | auxin-responsive protein, putative | 259773_at | 7.58* | 12.84 | 9.39* |
| AT1G77110 | auxin transport protein, putative | 264962_at | 52.91 | 84.81 | 90.87 |
| AT2G28350 | auxin-responsive factor (ARF10) | 265272_at | 8.18 | 10.94 | 7.46 |
| AT3G17600 | auxin-responsive protein, putative | 258352_at | 13.44 | 27.18 | 19.63 |
| AT3G44735 | phytosulfokines-related | 252624_at | 4.08* | 8.91 | 7.49 |
| AT4G24960 | ABA-responsive protein (HVA22d) | 254085_at | 1.80* | 6.08 | 13.88 |
| AT4G27260 | auxin-responsive GH3 family protein | 253908_at | 0.24* | 7.14 | 11.35 |
|  |  |  |  |  |  |
| *Membrane Trafficking* | |  | **ILN** | **MLN** | **MMN** |
| AT1G23220 | dynein light chain type 1 family protein | 264897_at | 4.36* | 15.05 | 18.45 |
| AT1G51640 | exocyst subunit EXO70 family protein | 256176_at | 4.84* | 23.67 | 14.00* |
| AT1G75170 /// AT5G04780 | SEC14 cytosolic factor family protein / phosphoglyceride transfer family protein | 256451_s_at | 2.02* | 10.58 | 8.20 |
| AT1G75370 | SEC14 cytosolic factor, putative / phosphatidylinositol transfer-like protein, putative | 261116_at | 2.80* | 7.42 | 6.73* |
| AT2G39380 | exocyst subunit EXO70 family protein | 266975_at | 5.28* | 11.41 | 5.38* |
| AT3G27000 | actin-related protein 2 (ARP2) | 257787_at | 2.40* | 4.63* | 4.06 |
|  |  |  |  |  |  |
| *Response to Biotic and Abiotic Factors, Defense* | | | **ILN** | **MLN** | **MMN** |
| AT1G22770 | gigantea protein (GI) | 264211_at | 3.91* | 4.26* | 5.51 |
| AT1G52690 | late embryogenesis abundant protein, putative / LEA protein, putative | 262128_at | 0.66* | 13.02* | 76.88 |
| AT1G74020 | strictosidine synthase family protein | 260391_at | 16.88 | 20.86 | 14.07* |
| AT2G45750 | dehydration-responsive family protein | 266920_at | 5.12* | 10.99 | 5.31* |
| AT3G25810 | myrcene/ocimene synthase, putative | 257637_at | 293.02 | 317.00 | 294.25 |
| AT4G31820 | phototropic-responsive NPH3 family protein | 253493_at | 4.36* | 16.49 | 17.36 |
| AT5G06720 | peroxidase, putative | 250646_at | 1.95* | 42.40 | 36.01 |
| AT5G24270 | calcineurin B-like protein, putative / calcium sensor homolog (SOS3) | 249783_at | 127.08 | 132.06 | 151.92 |
| AT5G38120 | 4-coumarate--CoA ligase family protein / 4-coumaroyl-CoA synthase family protein | 249540_at | 38.12 | 51.34 | 55.60 |
| AT5G44630 | terpene synthase/cyclase family protein | 249005_at | 39.25 | 39.36 | 44.72 |
| AT5G45050 | disease resistance protein-related | 248973_at | 2.19* | 4.77 | 3.42* |
| AT5G47800 | phototropic-responsive NPH3 family protein | 248772_at | 2.04* | 4.45* | 7.46 |
| AT5G63860 | UVB-resistance protein (UVR8) | 247307_at | 10.30 | 1.46* | 1.68* |
| AT5G67440 | phototropic-responsive NPH3 family protein | 246999_at | 0.51* | 7.72 | 5.26* |
|  |  |  |  |  |  |
| *Other* |  |  | **ILN** | **MLN** | **MMN** |
| AT1G08630 | L-allo-threonine aldolase-related | 264777_at | 20.03 | 26.30 | 14.93 |
| AT1G10570 | Ulp1 protease family protein | 263260_at | 2.65* | 3.37* | 3.84 |
| AT1G17960 | threonyl-tRNA synthetase, putative / threonine--tRNA ligase, putative | 255893_at | 23.95 | 74.47 | 59.63 |
| AT1G21160 | eukaryotic translation initiation factor 2 family protein / eIF-2 family protein | 261447_at | 2.89* | 4.30* | 5.88 |
| AT1G22050 | ubiquitin family protein | 255951_at | 5.24* | 6.84 | 7.53 |
| AT1G23010 | multi-copper oxidase type I family protein | 264752_at | 15.38* | 26.07 | 26.49 |
| AT1G23200 | pectinesterase family protein | 264891_at | 20.52 | 44.69 | 42.21 |
| AT1G31910 | GHMP kinase family protein | 246286_at | 2.18* | 3.94 | 3.56* |
| AT1G32560 | late embryogenesis abundant group 1 domain-containing protein / LEA group 1 domain-containing protein | 256464_at | 4.68* | 8.46 | 4.51* |
| AT1G36770 /// AT3G28530 | gypsy-like retrotransposon family (Athila) | 256595_x_at | 10.57 | 48.73 | 53.37 |
| AT1G37150 | holocarboxylase synthetase 2 (HCS2.d) | 262032_at | 5.05 | 3.63* | 2.80* |
| AT1G48280 | hydroxyproline-rich glycoprotein family protein | 262250_at | 6.57 | 0.68* | 1.01* |
| AT1G53885 | senescence-associated protein-related | 262226_at | 14.90 | 6.49 | 10.08 |
| AT1G58050 /// AT1G58060 | helicase domain-containing protein | 245863_s_at | 4.09 | 3.96 | 4.33 |
| AT1G68050 /// AT5G23410 /// AT5G42730 | F-box family protein (FKF1) / adagio 3 (ADO3) | 259990_s_at | 5.50* | 7.50 | 7.24 |
| AT1G70660 | ubiquitin-conjugating enzyme family protein | 260180_at | 2.17* | 6.81 | 6.38 |
| AT1G74820 | cupin family protein | 262214_at | 2.46* | 6.20* | 405.88 |
| AT2G03530 | F-box family protein-related | 265713_at | 8.70 | 18.53 | 15.02 |
| AT2G11140 | copia-like retrotransposon family | 265978_at | 2.31* | 3.77* | 5.74 |
| AT2G18360 | hydrolase, alpha/beta fold family protein | 265341_at | 11.33* | 24.10 | 24.55 |
| AT2G18780 | F-box family protein | 266064_at | 3.27* | 5.21 | 4.23* |
| AT2G19900 | malate oxidoreductase, putative | 266690_at | 1.79* | 1.71* | 9.34 |
| AT2G19930 | RNA-dependent RNA polymerase family protein | 266689_at | 2.37* | 7.49 | 5.07* |
| AT2G26740 /// AT2G26750 | epoxide hydrolase, soluble (sEH) | 267607_s_at | 3.45* | 11.02 | 10.94 |
| AT2G27820 | prephenate dehydratase family protein | 266257_at | 4.65* | 8.60 | 10.93 |
| AT2G28830 | armadillo/beta-catenin repeat family protein / U-box domain-containing protein | 266230_at | 2.56* | 4.96 | 5.08 |
| AT2G35920 | helicase domain-containing protein | 263936_at | 20.79 | 38.39 | 27.05 |
| AT2G36090 | F-box family protein | 263283_at | 16.52* | 19.11* | 11.96 |
| AT2G36240 | pentatricopeptide (PPR) repeat-containing protein | 263958_at | 3.09* | 3.82 | 2.46* |
| AT2G38960 | endoplasmic reticulum oxidoreductin 1 (ERO1) family protein | 266186_at | 3.71* | 5.69 | 4.79 |
| AT2G39060 | nodulin MtN3 family protein | 266201_at | 174.09 | 193.32 | 179.31 |
| AT3G02280 | flavodoxin family protein | 259126_at | 7.67 | 20.07 | 15.77 |
| AT3G03440 | armadillo/beta-catenin repeat family protein | 259043_at | 2.99* | 13.71 | 15.07 |
| AT3G06310 | NADH-ubiquinone oxidoreductase 19 kDa subunit (NDUFA8) family protein | 258881_at | 3.30* | 4.27 | 5.22 |
| AT3G13640 | RNase L inhibitor protein, putative | 256780_at | 6.16* | 7.56* | 9.80 |
| AT3G15200 | pentatricopeptide (PPR) repeat-containing protein | 256884_at | 1.00* | 2.98* | 7.38 |
| AT3G19000 | oxidoreductase, 2OG-Fe(II) oxygenase family protein | 256892_at | 2.26* | 6.48 | 3.66* |
| AT3G21280 | ubiquitin-specific protease 7, putative (UBP7) | 258045_at | 2.90* | 5.95 | 5.08 |
| AT3G24503 | aldehyde dehydrogenase (ALDH1a) | 258140_at | 8.31* | 10.45 | 11.43 |
| AT3G28220 | meprin and TRAF homology domain-containing protein / MATH domain-containing protein | 256577_at | 15.73 | 18.69 | 26.61 |
| AT3G45770 | oxidoreductase, zinc-binding dehydrogenase family protein | 252542_at | 9.58 | 7.87 | 5.96 |
| AT3G48000 | aldehyde dehydrogenase (ALDH2) | 252372_at | 8.96 | 8.93 | 10.04 |
| AT3G49810 | U-box domain-containing protein | 252230_at | 2.68* | 5.23 | 4.72* |
| AT3G55890 | yippee family protein | 251743_at | 38.15 | 10.49 | 23.55 |
| AT3G62790 | NADH-ubiquinone oxidoreductase-related | 251186_at | 4.31 | 3.16* | 4.49 |
| AT4G04590 | transposable element gene | 255313_at | 1.13* | 8.50 | 2.64* |
| AT4G10610 | RNA-binding protein, putative | 254990_at | 2.35* | 3.41* | 4.14 |
| AT4G13460 | SET domain-containing protein (SUVH9) | 254745_at | 1.01* | 1.59* | 4.06 |
| AT4G17670 | senescence-associated protein-related | 245401_at | 3.55* | 8.07 | 6.39 |
| AT4G19850 | lectin-related | 254546_at | 1.61* | 3.56* | 13.63 |
| AT4G22150 | UBX domain-containing protein | 254348_at | 4.25* | 4.64* | 5.69 |
| AT4G22753 | sterol desaturase family protein | 254333_at | 11.85 | 3.24* | 2.69* |
| AT4G25300 /// AT4G25310 | oxidoreductase, 2OG-Fe(II) oxygenase family protein | 254053_s_at | 1.57* | 16.70 | 10.68 |
| AT5G03140 | lectin protein kinase family protein | 250981_at | 1.44* | 5.66 | 3.16* |
| AT5G17980 | C2 domain-containing protein | 250070_at | 3.13* | 3.65* | 5.71 |
| AT5G19660 | subtilase family protein | 245960_at | 2.36* | 4.12 | 3.03* |
| AT5G19730 | pectinesterase family protein | 245965_at | 7.61* | 27.04 | 25.17 |
| AT5G24580 | copper-binding family protein | 249755_at | 1.52* | 12.69 | 9.82* |
| AT5G38130 | transferase family protein | 249541_at | 16.75* | 70.03 | 57.07 |
| AT5G41220 /// AT5G41240 | glutathione S-transferase, putative | 249316_s_at | 3.89* | 4.59 | 4.66 |
| AT5G42230 | serine carboxypeptidase S10 family protein | 249241_at | 0.71* | 53.38 | 34.80 |
| AT5G43580 | protease inhibitor, putative | 249101_at | 1.22* | 17.08* | 29.27 |
| AT5G44620 | cytochrome P450 family protein | 249012_at | 19.47 | 23.88 | 23.82 |
| AT5G44730 | haloacid dehalogenase-like hydrolase family protein | 249015_at | 5.42* | 16.36 | 15.37 |
| AT5G47060 | senescence-associated protein-related | 248820_at | 15.25 | 15.56 | 15.84 |
| AT5G48950 | thioesterase family protein | 248627_at | 6.75* | 10.77 | 7.94 |
| AT5G52330 | meprin and TRAF homology domain-containing protein / MATH domain-containing protein | 248354_at | 1.23* | 8.28 | 7.55* |
| AT5G58660 | oxidoreductase, 2OG-Fe(II) oxygenase family protein | 247774_at | 4.71* | 20.61 | 19.16 |
| AT5G60760 | 2-phosphoglycerate kinase-related | 247582_at | 26.79 | 30.25 | 26.13 |
|  |  |  |  |  |  |
| *Uncharacterized/Hypothetical Proteins* | |  | **ILN** | **MLN** | **MMN** |
| AT1G11440 | expressed protein | 261871_at | 2.56* | 6.80 | 6.72 |
| AT1G13940 | expressed protein | 262663_at | 2.12* | 3.39* | 4.28 |
| AT1G17490 | expressed protein | 261038_at | 5.31* | 6.00 | 5.42 |
| AT1G18510 | hypothetical protein | 261714_at | 4.95* | 15.63 | 8.00 |
| AT1G18720 | expressed protein | 261379_at | 7.20* | 9.00 | 8.19 |
| AT1G20070 | expressed protein | 261247_at | 0.11* | 4.64* | 17.96 |
| AT1G21770 | expressed protein | 262499_at | 4.28* | 7.77 | 6.80* |
| AT1G26620 | expressed protein | 261028_at | 3.07* | 4.94* | 5.97 |
| AT1G27870 /// AT2G16100 | hypothetical protein | 257455_s_at | 3.98* | 5.79* | 7.86 |
| AT1G28410 | expressed protein | 261495_at | 2.86* | 5.16 | 7.78 |
| AT1G32290 | hypothetical protein | 260689_at | 7.20* | 12.62 | 5.81 |
| AT1G36550 | hypothetical protein | 261960_at | 2.27* | 2.99* | 5.33 |
| AT1G54200 | expressed protein | 263002_at | 4.58* | 11.82 | 6.76* |
| AT1G55830 | expressed protein | 264542_at | 2.47* | 3.45* | 3.78 |
| AT1G56320 | expressed protein | 256217_at | 1.38* | 9.84 | 4.77* |
| AT1G64405 | expressed protein | 259735_at | 16.66 | 14.04 | 17.76 |
| AT1G66110 | hypothetical protein | 256519_at | 3.24* | 8.39 | 5.54* |
| AT1G68650 | expressed protein | 262277_at | 2.13* | 6.23 | 4.51* |
| AT1G69360 | expressed protein | 260361_at | 3.41* | 7.28 | 7.69 |
| AT1G70270 | expressed protein | 264341_at | 28.32 | 29.76 | 44.74 |
| AT1G75730 | expressed protein | 262967_at | 9.85 | 8.90 | 10.79 |
| AT2G03540 | expressed protein | 265709_at | 1.77* | 5.37* | 9.28 |
| AT2G06390 | hypothetical protein | 263769_at | 5.76* | 6.55* | 12.33 |
| AT2G07721 | hypothetical protein | 265234_at | 2.51* | 3.36* | 5.42 |
| AT2G10560 /// AT5G04860 | hypothetical protein | 246982_s_at | 1.56* | 7.70* | 14.54 |
| AT2G19850 /// AT3G54730 | hypothetical protein | 266680_s_at | 2.26* | 12.94 | 25.85 |
| AT2G25720 | expressed protein | 265909_at | 4.22* | 5.91 | 5.57 |
| AT2G35820 | expressed protein | 263947_at | 5.71 | 3.42* | 3.37* |
| AT2G39080 | expressed protein | 266192_at | 7.79 | 13.29 | 9.09 |
| AT2G42570 | expressed protein | 263496_at | 10.55 | 2.86* | 3.49* |
| AT3G05570 | expressed protein | 259110_at | 6.09 | 3.96* | 4.88* |
| AT3G06590 | expressed protein | 258511_at | 6.35* | 10.89 | 10.63 |
| AT3G06600 | expressed protein | 258510_at | 2.57* | 6.24* | 13.45 |
| AT3G13845 | expressed protein | 257609_at | 6.72 | 2.98* | 4.01* |
| AT3G13910 | expressed protein | 258201_at | 3.17* | 10.20 | 10.15 |
| AT3G25640 | expressed protein | 256759_at | 1.30* | 13.31 | 23.07 |
| AT3G25805 | expressed protein | 257647_at | 8.97 | 7.19 | 6.77* |
| AT3G27320 | expressed protein | 257145_at | 3.85* | 4.31* | 4.60 |
| AT3G27340 | expressed protein | 257741_at | 4.57 | 2.83* | 1.83* |
| AT3G30560 | hypothetical protein | 258307_x_at | 2.59* | 2.63* | 4.80 |
| AT3G50550 | expressed protein | 252165_at | 3.42* | 5.38 | 4.42* |
| AT3G51400 | expressed protein | 252118_at | 9.78* | 16.10 | 21.47 |
| AT3G51940 | expressed protein | 252082_at | 3.27* | 4.35 | 3.58* |
| AT3G55910 | expressed protein | 251766_at | 52.56 | 14.36 | 24.97 |
| AT3G60780 | expressed protein | 251339_at | 1.58* | 27.46 | 21.16 |
| AT4G00530 | hypothetical protein | 255680_at | 4.30 | 1.84* | 1.14* |
| AT4G01897 | expressed protein | 255554_at | 3.48* | 8.53 | 4.78 |
| AT4G03180 | expressed protein | 255434_at | 3.80* | 5.39 | 4.93 |
| AT4G09640 | expressed protein | 255043_at | 1.56* | 6.62 | 4.33* |
| AT4G24130 | expressed protein | 254201_at | 3.54* | 11.61 | 11.33* |
| AT4G27660 | expressed protein | 253883_at | 0.90* | 6.38 | 6.05* |
| AT4G31570 | expressed protein | 253532_at | 7.86 | 13.09 | 15.41 |
| AT4G35240 | expressed protein | 253185_at | 5.98* | 7.08 | 6.83 |
| AT4G35990 | hypothetical protein | 253122_at | 7.73 | 8.52 | 8.32 |
| AT5G03130 | hypothetical protein | 250980_at | 1.78* | 8.31 | 6.86 |
| AT5G14150 | expressed protein | 250224_at | 4.41* | 11.91 | 7.22* |
| AT5G15320 | expressed protein | 250168_at | 5.17 | 2.83* | 2.94* |
| AT5G23380 | expressed protein | 249824_at | 5.74* | 11.15* | 12.02 |
| AT5G26731 | expressed protein | 246842_at | 4.59* | 16.81 | 20.10 |
| AT5G27210 | expressed protein | 246793_at | 3.70* | 5.62 | 4.65* |
| AT5G35880 | hypothetical protein | 249669_at | 4.53 | 5.72 | 6.41 |
| AT5G46115 | expressed protein | 248887_at | 5.16* | 24.28 | 27.24 |
| AT5G52870 | expressed protein | 248286_at | 1.26* | 4.38* | 5.98 |
| AT5G53800 | expressed protein | 248228_at | 4.89 | 5.35 | 4.71* |
| AT5G53900 | expressed protein | 248238_at | 6.92* | 5.42* | 8.82 |
| AT5G54850 | expressed protein | 248133_at | 1.47* | 5.43* | 5.23 |
| AT5G55010 | hypothetical protein | 248141_at | 9.81* | 11.66* | 14.70 |
| AT5G63905 | expressed protein | 247305_at | 3.21* | 6.26 | 10.09 |
| ^a^ All genes displaying a 3-fold or greater change in normalized probe signal intensity in one or more nectary types (MLN, MMN, ILN) over all individual reference tissues are displayed (t-test p-value cutoff 0.05 and FDR q-value cutoff 0.1; data summarized in Table 2). Signal values not meeting these criteria within specific nectary type(s) are indicated with an asterisk. Full normalized probe intensities and p and q-values for individual nectary samples are available in Additional files 4, 5, and 6. | | | | | |
