# Supplementary material for: Uncovering the Arabidopsis thaliana nectary transcriptome: investigation of differential gene expression in floral nectariferous tissues
Source: BMC Plant Biol. 2009 Jul 15;9:92. doi: 10.1186/1471-2229-9-92 (PMC2720969; doi:10.1186/1471-2229-9-92)
Supplement: Additional file 9 — Genes displaying differential expression between mature and immature lateral nectaries. All genes displaying a 5-fold or greater difference in probe signal value between MLN and ILN are shown (t test p-value cutoff 0.05, and FDR q-value cutoff 0.05). [file 1471-2229-9-92-S9.doc]

| **Additional file 9 - Genes displaying differential expression between mature and immature lateral nectaries** | | | | |
| --- | --- | --- | --- | --- |
| Locus | TAIR Annotation | Probe Set | MLN^a^  ILN | MLN  MMN |
| *Transcription Factors* | |  |  |  |
| AT1G02340 | long hypocotyl in far-red 1 (HFR1) / reduced phytochrome signaling (REP1) / basic helix-loop-helix FBI1 protein (FBI1) | 259417_at | 5.6 | 0.84 |
| AT1G15100 | zinc finger (C3HC4-type RING finger) family protein | 262590_at | 7.36 | 0.6 |
| AT1G18400 | basic helix-loop-helix (bHLH) family protein | 261717_at | 6.64 | 1.27 |
| AT1G65920 | regulator of chromosome condensation (RCC1) family protein / zinc finger protein-related | 261917_at | 0.17 | 0.59 |
| AT2G14210 | MADS-box protein (ANR1) | 263295_at | 5.81 | 1.36 |
| AT2G16720 | myb family transcription factor | 265359_at | 5.87 | 5.36 |
| AT2G21320 | zinc finger (B-box type) family protein | 263739_at | 7.76 | 0.94 |
| AT2G22850 | bZIP transcription factor family protein | 266798_at | 7.12 | 2.05 |
| AT2G24570 | WRKY family transcription factor | 263797_at | 6.55 | 1.14 |
| AT2G46830 | myb-related transcription factor (CCA1) | 266719_at | 6.71 | 1.23 |
| AT3G01470 | homeobox-leucine zipper protein 5 (HAT5) / HD-ZIP protein 5 / HD-ZIP protein (HB-1) | 259165_at | 10.23 | 1.78 |
| AT3G01530 | myb family transcription factor (MYB57) | 259187_at | 6.4 | 1.21 |
| AT3G06330 | zinc finger (C3HC4-type RING finger) family protein | 258882_at | 6.14 | 1.17 |
| AT3G09600 | myb family transcription factor | 258724_at | 6.34 | 1.76 |
| AT3G47500 | Dof-type zinc finger domain-containing protein | 252429_at | 8.24 | 1.4 |
| AT3G50410 | Dof-type zinc finger domain-containing protein | 252210_at | 11.49 | 2.27 |
| AT4G12080 | DNA-binding family protein | 254853_at | 8.82 | 3 |
| AT4G34990 | myb family transcription factor (MYB32) | 253219_at | 5.32 | 1.02 |
| AT4G36710 | scarecrow transcription factor family protein | 246230_at | 6.56 | 1.37 |
| AT4G37890 | zinc finger (C3HC4-type RING finger) family protein | 253011_at | 0.12 | 0.87 |
| AT4G38960 | zinc finger (B-box type) family protein | 252917_at | 7.94 | 1.16 |
| AT5G07680 | no apical meristem (NAM) family protein | 250597_at | 38.65 | 1.65 |
| AT5G10030 | bZIP family transcription factor (OBF4) | 250463_at | 9.12 | 2.5 |
| AT5G35970 | DNA-binding protein, putative | 249677_at | 5.12 | 0.72 |
| AT5G40350 | myb family transcription factor (MYB24) | 249349_at | 0.19 | 0.84 |
| AT5G40360 | myb family transcription factor (MYB115) | 249350_at | 40.92 | 0.72 |
| AT5G52830 | WRKY family transcription factor | 248306_at | 6.53 | 1.52 |
| AT5G61430 | no apical meristem (NAM) family protein | 247519_at | 7.71 | 2.88 |
|  |  |  |  |  |
| *Carbohydrate Metabolism* | |  | MLN^a^  ILN | MLN  MMN |
| AT1G05170 | galactosyltransferase family protein | 264583_at | 8.54 | 1.05 |
| AT1G69200 | pfkB-type carbohydrate kinase family protein | 260343_at | 0.19 | 0.67 |
| AT2G31750 | UDP-glucoronosyl/UDP-glucosyl transferase family protein | 263473_at | 0.15 | 0.58 |
| AT2G44460 | glycosyl hydrolase family 1 protein | 267389_at | 17.33 | 4.75 |
| AT2G44480 | glycosyl hydrolase family 1 protein | 267391_at | 47.38 | 2.7 |
| AT3G23920 | beta-amylase, putative / 1,4-alpha-D-glucan maltohydrolase, putative | 256861_at | 7.28 | 0.67 |
| AT3G54090 | pfkB-type carbohydrate kinase family protein | 251935_at | 0.18 | 0.98 |
| AT3G57520 | alkaline alpha galactosidase, putative | 251642_at | 0.15 | 0.74 |
| AT4G34131 /// AT4G34135 | UDP-glucoronosyl/UDP-glucosyl transferase family protein | 253268_s_at | 21.29 | 0.88 |
| AT5G08380 | alpha-galactosidase, putative / melibiase, putative / alpha-D-galactoside galactohydrolase, putative | 246055_at | 0.12 | 0.88 |
| AT5G25980 /// AT5G26000 | glycosyl hydrolase family 1 protein | 246880_s_at | 0.09 | 0.2 |
| AT5G49360 | glycosyl hydrolase family 3 protein | 248622_at | 0.05 | 0.97 |
| AT5G51950 | glucose-methanol-choline (GMC) oxidoreductase family protein | 248368_at | 0.1 | 0.56 |
| AT5G65140 | trehalose-6-phosphate phosphatase, putative | 247228_at | 6.92 | 0.67 |
|  |  |  |  |  |
| *Lipid Metabolism & Processing* | |  | MLN^a^  ILN | MLN  MMN |
| AT1G12090 | protease inhibitor/seed storage/lipid transfer protein (LTP) family protein | 264371_at | 0.16 | 1.26 |
| AT1G31550 | GDSL-motif lipase, putative | 256489_at | 0.16 | 0.93 |
| AT1G55260 | protease inhibitor/seed storage/lipid transfer protein (LTP) family protein | 259660_at | 0.05 | 0.47 |
| AT1G66120 | acyl-activating enzyme 11 (AAE11) | 256521_at | 17.2 | 1.22 |
| AT1G70670 /// AT1G70680 | caleosin-related family protein | 260208_s_at | 10.54 | 1.98 |
| AT1G75900 | family II extracellular lipase 3 (EXL3) | 262682_at | 0.04 | 0.29 |
| AT3G11430 | phospholipid/glycerol acyltransferase family protein | 259282_at | 76.21 | 1.39 |
| AT3G16370 | GDSL-motif lipase/hydrolase family protein | 259375_at | 0.16 | 0.68 |
| AT3G62860 | esterase/lipase/thioesterase family protein | 251235_at | 0.15 | 0.48 |
| AT4G30140 | GDSL-motif lipase/hydrolase family protein | 253660_at | 60.23 | 1.22 |
| AT5G45950 | GDSL-motif lipase/hydrolase family protein | 248921_at | 5.1 | 1.06 |
|  |  |  |  |  |
| *Transporter and Channel Proteins* | |  | MLN^a^  ILN | MLN  MMN |
| AT1G01340 | cyclic nucleotide-regulated ion channel (CNGC10) (ACBK1) | 261027_at | 7.41 | 1.32 |
| AT1G15500 /// AT1G80300 | chloroplast ADP, ATP carrier protein, putative / ADP, ATP translocase, putative / adenine nucleotide translocase, putative | 261767_s_at | 0.17 | 0.56 |
| AT1G51340 | MATE efflux family protein | 265151_at | 6.05 | 1.15 |
| AT1G69860 | proton-dependent oligopeptide transport (POT) family protein | 260400_at | 6.98 | 2.62 |
| AT1G69870 | proton-dependent oligopeptide transport (POT) family protein | 260410_at | 11.72 | 0.84 |
| AT1G71330 /// AT3G13080 | ABC transporter family protein | 259937_s_at | 6.68 | 1.13 |
| AT1G78000 | sulfate transporter (Sultr1;2) | 262133_at | 8.2 | 3.45 |
| AT2G28900 | mitochondrial import inner membrane translocase subunit Tim17/Tim22/Tim23 family protein | 266225_at | 0.17 | 0.42 |
| AT2G29530 | mitochondrial import inner membrane translocase (TIM10) | 266239_at | 0.1 | 0.46 |
| AT2G39350 | ABC transporter family protein | 267008_at | 14 | 0.41 |
| AT2G41560 | calcium-transporting ATPase 4, plasma membrane-type / Ca2+-ATPase, isoform 4 (ACA4) | 245117_at | 0.2 | 0.97 |
| AT3G46560 | mitochondrial import inner membrane translocase (TIM9) | 252479_at | 0.15 | 0.75 |
| AT3G47730 | ABC transporter family protein | 252379_at | 5.96 | 1.58 |
| AT3G55110 | ABC transporter family protein | 251826_at | 0.12 | 0.76 |
| AT3G62150 | multidrug resistant (MDR) ABC transporter, putative | 251248_at | 6.77 | 1.45 |
| AT5G14940 | proton-dependent oligopeptide transport (POT) family protein | 246566_at | 6.35 | 0.12 |
| AT5G46050 | proton-dependent oligopeptide transport (POT) family protein | 248932_at | 0.16 | 1.51 |
| AT5G52280 | protein transport protein-related | 248344_at | 0.14 | 0.57 |
| AT5G62680 | proton-dependent oligopeptide transport (POT) family protein | 247440_at | 9.48 | 1.43 |
|  |  |  |  |  |
| *Signaling and Development* | |  | MLN^a^  ILN | MLN  MMN |
| AT1G02190 | CER1 protein, putative | 264180_at | 0.2 | 1.09 |
| AT1G02205 | CER1 protein | 264146_at | 0.18 | 0.53 |
| AT1G32170 | xyloglucan:xyloglucosyl transferase, putative / xyloglucan endotransglycosylase, putative / endo-xyloglucan transferase, putative (XTR4) | 245794_at | 5.37 | 1 |
| AT1G66150 | leucine-rich repeat protein kinase, putative (TMK1) | 256516_at | 0.14 | 0.9 |
| AT1G67720 | leucine-rich repeat family protein / protein kinase family protein | 245202_at | 0.11 | 0.31 |
| AT1G69040 | ACT domain containing protein (ACR4) | 257516_at | 0.1 | 0.46 |
| AT2G02780 | leucine-rich repeat transmembrane protein kinase, putative | 267481_at | 0.17 | 0.62 |
| AT2G03090 | expansin, putative (EXP15) | 266770_at | 0.18 | 1.03 |
| AT2G06850 | xyloglucan:xyloglucosyl transferase / xyloglucan endotransglycosylase / endo-xyloglucan transferase (EXT) (EXGT-A1) | 266215_at | 5.77 | 0.85 |
| AT2G26440 | pectinesterase family protein | 245052_at | 0.06 | 1.74 |
| AT2G28110 | exostosin family protein | 266156_at | 7.46 | 1.57 |
| AT2G28950 | expansin, putative (EXP6) | 266790_at | 0.08 | 0.52 |
| AT2G40120 | protein kinase family protein | 263383_at | 17.1 | 1.17 |
| AT3G04910 | protein kinase family protein | 259080_at | 0.14 | 0.81 |
| AT3G05640 | protein phosphatase 2C, putative / PP2C, putative | 258901_at | 6.84 | 0.47 |
| AT3G09150 | phytochromobilin:ferredoxin oxidoreductase, chloroplast / phytochromobilin synthase (HY2) | 259210_at | 0.2 | 0.8 |
| AT3G14310 | pectinesterase family protein | 258369_at | 0.17 | 0.95 |
| AT3G19820 | cell elongation protein / DWARF1 / DIMINUTO (DIM) | 257938_at | 0.13 | 1.28 |
| AT3G50070 | cyclin family protein | 252189_at | 6.38 | 1.46 |
| AT4G30270 | MERI-5 protein (MERI-5) (MERI5B) / endo-xyloglucan transferase / xyloglucan endo-1,4-beta-D-glucanase (SEN4) | 253666_at | 66.21 | 0.93 |
| AT4G38810 | calcium-binding EF hand family protein | 252915_at | 18.85 | 0.51 |
| AT5G50120 | transducin family protein / WD-40 repeat family protein | 248535_at | 5.73 | 1.4 |
|  |  |  |  |  |
| *Hormone Metabolism and Response* | |  | MLN^a^  ILN | MLN  MMN |
| AT1G02400 | gibberellin 2-oxidase, putative / GA2-oxidase, putative | 259445_at | 20.46 | 0.53 |
| AT1G19050 | two-component responsive regulator / response regulator 7 (ARR7) | 259466_at | 17.25 | 0.61 |
| AT1G66350 | gibberellin regulatory protein (RGL1) | 260141_at | 0.1 | 0.83 |
| AT2G17500 | auxin efflux carrier family protein | 263073_at | 5.09 | 1.83 |
| AT3G07390 | auxin-responsive protein / auxin-induced protein (AIR12) | 259018_at | 0.03 | 0.47 |
| AT3G49780 | phytosulfokines 3 (PSK3) | 252234_at | 7.15 | 0.8 |
| AT3G61900 | auxin-responsive family protein | 251291_at | 15.55 | 2.05 |
| AT4G12410 | auxin-responsive family protein | 254809_at | 8.34 | 1.68 |
| AT4G27260 | auxin-responsive GH3 family protein | 253908_at | 29.71 | 0.63 |
| AT4G36110 | auxin-responsive protein, putative | 253103_at | 27.58 | 1.4 |
| AT5G51810 | gibberellin 20-oxidase, putative | 248371_at | 8.41 | 0.64 |
| AT5G62920 | two-component responsive regulator / response regulator 6 (ARR6) | 247406_at | 6.93 | 0.59 |
|  |  |  |  |  |
| *Membrane Trafficking* | |  | MLN^a^  ILN | MLN  MMN |
| AT1G30900 | vacuolar sorting receptor, putative | 265161_at | 40.93 | 1.93 |
| AT1G75170 /// AT5G04780 | SEC14 cytosolic factor family protein / phosphoglyceride transfer family protein | 256451_s_at | 5.23 | 1.29 |
|  |  |  |  |  |
| *Response to Biotic and Abiotic Factors, Defense* | |  | MLN^a^  ILN | MLN  MMN |
| AT1G07610 | metallothionein-like protein 1C (MT-1C) | 261410_at | 19.67 | 0.69 |
| AT1G20440 | dehydrin (COR47) | 259570_at | 20.8 | 0.67 |
| AT1G20450 | dehydrin (ERD10) | 259516_at | 5.56 | 0.86 |
| AT1G24020 | Bet v I allergen family protein | 263034_at | 0.12 | 0.44 |
| AT1G52690 | late embryogenesis abundant protein, putative / LEA protein, putative | 262128_at | 19.7 | 0.17 |
| AT1G65690 | harpin-induced protein-related / HIN1-related / harpin-responsive protein-related | 262930_at | 12.23 | 1.06 |
| AT1G66100 | thionin, putative | 256527_at | 6.35 | 0.48 |
| AT1G73680 | pathogen-responsive alpha-dioxygenase, putative | 260060_at | 7.24 | 2.43 |
| AT1G76180 | dehydrin (ERD14) | 261749_at | 5.21 | 0.57 |
| AT1G77760 | nitrate reductase 1 (NR1) | 259681_at | 5.41 | 0.58 |
| AT1G78780 | pathogenesis-related family protein | 264301_at | 17.45 | 1.48 |
| AT2G18950 | homogentisate phytylprenyltransferase family protein (HPT1) / tocopherol phytyltransferase family protein (TPT1) | 266938_at | 0.2 | 0.75 |
| AT2G37040 | phenylalanine ammonia-lyase 1 (PAL1) | 263845_at | 14.34 | 0.57 |
| AT3G05880 | hydrophobic protein (RCI2A) / low temperature and salt responsive protein (LTI6A) | 258735_at | 0.19 | 0.34 |
| AT3G10340 | phenylalanine ammonia-lyase, putative | 259149_at | 8.94 | 1.07 |
| AT3G10985 | wound-responsive protein-related | 256433_at | 5.76 | 0.58 |
| AT3G11660 | harpin-induced family protein / HIN1 family protein / harpin-responsive family protein | 259102_at | 13.67 | 0.33 |
| AT3G12580 | heat shock protein 70, putative / HSP70, putative | 256245_at | 6.93 | 1.17 |
| AT3G22840 | chlorophyll A-B binding family protein / early light-induced protein (ELIP) | 258321_at | 13.65 | 0.45 |
| AT3G26740 | light responsive protein-related | 257832_at | 0.2 | 0.44 |
| AT3G48090 | disease resistance protein (EDS1) | 252373_at | 7.94 | 0.71 |
| AT3G53260 | phenylalanine ammonia-lyase 2 (PAL2) | 251984_at | 8.06 | 0.44 |
| AT4G01410 | harpin-induced family protein / HIN1 family protein / harpin-responsive family protein | 255577_at | 0.12 | 1.05 |
| AT4G12400 | stress-inducible protein, putative | 254839_at | 6.43 | 1.26 |
| AT4G15910 | drought-responsive protein / drought-induced protein (Di21) | 245523_at | 0.08 | 0.76 |
| AT4G31870 | glutathione peroxidase, putative | 253496_at | 6.22 | 0.7 |
| AT4G37760 | squalene monooxygenase, putative / squalene epoxidase, putative | 253039_at | 7.68 | 0.87 |
| AT4G37910 | heat shock protein 70, mitochondrial, putative / HSP70, mitochondrial, putative | 253013_at | 0.17 | 0.63 |
| AT5G06720 | peroxidase, putative | 250646_at | 21.74 | 1.18 |
| AT5G06870 | polygalacturonase inhibiting protein 2 (PGIP2) | 250669_at | 0.18 | 2.78 |
| AT5G23310 | superoxide dismutase (Fe) / iron superoxide dismutase 3 (FSD3) | 249826_at | 0.14 | 0.77 |
| AT5G38710 | proline oxidase, putative / osmotic stress-responsive proline dehydrogenase, putative | 249527_at | 9.08 | 1.29 |
| AT5G40170 | disease resistance family protein | 249393_at | 5.23 | 1.08 |
| AT5G52390 | photoassimilate-responsive protein, putative | 248333_at | 5.34 | 0.37 |
| AT5G54660 | heat shock protein-related | 248172_at | 6.88 | 4.81 |
| AT5G63860 | UVB-resistance protein (UVR8) | 247307_at | 0.14 | 0.87 |
| AT5G64260 | phosphate-responsive protein, putative | 247280_at | 10.38 | 0.41 |
| AT5G67440 | phototropic-responsive NPH3 family protein | 246999_at | 15.03 | 1.47 |
|  |  |  |  |  |
| *Gene Expression, Protein Synthesis* | |  | MLN^a^  ILN | MLN  MMN |
| AT1G05190 | ribosomal protein L6 family protein | 264575_at | 0.11 | 0.87 |
| AT1G07320 | 50S ribosomal protein L4, chloroplast (CL4) | 261078_at | 0.1 | 0.78 |
| AT1G09200 | histone H3 | 264262_at | 0.14 | 0.75 |
| AT1G35680 | 50S ribosomal protein L21, chloroplast / CL21 (RPL21) | 262029_at | 0.12 | 0.73 |
| AT1G55490 | RuBisCO subunit binding-protein beta subunit, chloroplast / 60 kDa chaperonin beta subunit / CPN-60 beta | 265076_at | 0.17 | 0.89 |
| AT1G59990 | DEAD/DEAH box helicase, putative (RH22) | 263679_at | 0.19 | 0.83 |
| AT1G64880 | ribosomal protein S5 family protein | 262880_at | 0.14 | 0.7 |
| AT1G77750 | 30S ribosomal protein S13, chloroplast, putative | 259678_at | 0.13 | 0.56 |
| AT2G18400 | ribosomal protein L6 family protein | 265338_at | 0.17 | 0.79 |
| AT2G19750 | 40S ribosomal protein S30 (RPS30A) | 266705_at | 0.14 | 0.76 |
| AT2G21790 | ribonucleoside-diphosphate reductase small chain, putative / ribonucleotide reductase, putative | 263882_at | 0.11 | 0.63 |
| AT2G29570 | proliferating cell nuclear antigen 2 (PCNA2) | 266297_at | 0.18 | 0.79 |
| AT2G33210 | chaperonin, putative | 245164_at | 0.19 | 0.88 |
| AT2G38140 | chloroplast 30S ribosomal protein S31 (PSRP4) | 267088_at | 0.13 | 0.79 |
| AT2G38810 | histone H2A, putative | 263264_at | 0.19 | 0.99 |
| AT2G43030 | ribosomal protein L3 family protein | 265247_at | 0.16 | 0.86 |
| AT3G04920 | 40S ribosomal protein S24 (RPS24A) | 259090_at | 0.12 | 0.91 |
| AT3G13470 | chaperonin, putative | 256983_at | 0.19 | 1.23 |
| AT3G18760 | ribosomal protein S6 family protein | 257755_at | 0.13 | 0.58 |
| AT3G20670 | histone H2A, putative | 256666_at | 0.18 | 0.89 |
| AT3G23990 | chaperonin (CPN60) (HSP60) | 256905_at | 0.1 | 0.89 |
| AT3G25920 | 50S ribosomal protein L15, chloroplast (CL15) | 258076_at | 0.11 | 0.89 |
| AT3G27060 | ribonucleoside-diphosphate reductase small chain, putative / ribonucleotide reductase, putative | 257809_at | 0.17 | 0.97 |
| AT3G27360 | histone H3 | 257714_at | 0.11 | 0.74 |
| AT3G28900 | 60S ribosomal protein L34 (RPL34C) | 257141_at | 0.16 | 0.97 |
| AT3G44890 | 50S ribosomal protein L9, chloroplast (CL9) | 246339_at | 0.14 | 0.67 |
| AT3G49470 | nascent polypeptide-associated complex (NAC) domain-containing protein | 252277_at | 0.18 | 0.64 |
| AT3G52150 | RNA recognition motif (RRM)-containing protein | 252032_at | 0.1 | 0.74 |
| AT3G55400 | methionyl-tRNA synthetase / methionine--tRNA ligase / MetRS (cpMetRS) | 251807_at | 0.2 | 0.99 |
| AT4G01310 | ribosomal protein L5 family protein | 255623_at | 0.07 | 0.71 |
| AT4G02930 | elongation factor Tu, putative / EF-Tu, putative | 255455_at | 0.14 | 0.7 |
| AT4G17560 | ribosomal protein L19 family protein | 245357_at | 0.08 | 1.04 |
| AT4G24770 | 31 kDa ribonucleoprotein, chloroplast, putative / RNA-binding protein RNP-T, putative / RNA-binding protein 1/2/3, putative / RNA-binding protein cp31, putative | 254126_at | 0.16 | 0.78 |
| AT4G29060 | elongation factor Ts family protein | 253758_at | 0.14 | 0.72 |
| AT4G30330 | small nuclear ribonucleoprotein E, putative / snRNP-E, putative / Sm protein E, putative | 253607_at | 0.16 | 0.84 |
| AT4G34620 | ribosomal protein S16 family protein | 253201_at | 0.16 | 0.79 |
| AT5G10390 | histone H3 | 250434_at | 0.12 | 0.76 |
| AT5G10400 | histone H3 | 250433_at | 0.12 | 0.81 |
| AT5G17870 | plastid-specific ribosomal protein-related | 250058_at | 0.18 | 0.78 |
| AT5G22440 | 60S ribosomal protein L10A (RPL10aC) | 249945_at | 0.14 | 0.87 |
| AT5G22880 | histone H2B, putative | 249916_at | 0.12 | 1.04 |
| AT5G24120 | RNA polymerase sigma subunit SigE (sigE) / sigma-like factor (SIG5) | 249769_at | 5.23 | 0.78 |
| AT5G39800 /// AT5G40080 | 60S ribosomal protein-related | 249424_s_at | 0.19 | 0.65 |
| AT5G46160 | ribosomal protein L14 family protein / huellenlos paralog (HLP) | 248878_at | 0.19 | 1.22 |
| AT5G47190 | ribosomal protein L19 family protein | 248798_at | 0.13 | 0.97 |
| AT5G59690 /// AT5G59970 | histone H4 | 247692_s_at | 0.17 | 1.04 |
| AT5G59870 | histone H2A, putative | 247651_at | 0.14 | 0.92 |
| AT5G65220 | ribosomal protein L29 family protein | 247201_at | 0.19 | 0.72 |
| AT5G65360 | histone H3 | 247192_at | 0.19 | 0.81 |
|  |  |  |  |  |
| *Other* | |  | MLN^a^  ILN | MLN  MMN |
| AT1G01300 | aspartyl protease family protein | 261055_at | 0.18 | 1.23 |
| AT1G02920 /// AT1G02930 | glutathione S-transferase, putative | 262119_s_at | 6.72 | 0.42 |
| AT1G06430 | FtsH protease, putative | 262626_at | 9.53 | 1.18 |
| AT1G06570 | 4-hydroxyphenylpyruvate dioxygenase (HPD) | 262635_at | 5.46 | 1.03 |
| AT1G12140 | flavin-containing monooxygenase family protein / FMO family protein | 260993_at | 6.9 | 0.82 |
| AT1G13270 | metallopeptidase M24 family protein | 259363_at | 0.19 | 0.84 |
| AT1G21440 | mutase family protein | 260902_at | 0.11 | 0.34 |
| AT1G35190 | oxidoreductase, 2OG-Fe(II) oxygenase family protein | 245756_at | 62.26 | 0.94 |
| AT1G48280 | hydroxyproline-rich glycoprotein family protein | 262250_at | 0.1 | 0.68 |
| AT1G57590 | pectinacetylesterase, putative | 246403_at | 7.76 | 0.45 |
| AT1G61870 | pentatricopeptide (PPR) repeat-containing protein | 264286_at | 0.2 | 0.86 |
| AT1G62900 /// AT1G63140 | O-methyltransferase, putative | 261103_s_at | 5.9 | 0.86 |
| AT1G64940 /// AT1G64950 /// AT2G12190 | cytochrome P450, putative | 266155_at | 6.08 | 1.19 |
| AT1G65370 | meprin and TRAF homology domain-containing protein / MATH domain-containing protein | 264166_at | 0.19 | 1.09 |
| AT1G77060 | mutase family protein | 264954_at | 0.12 | 0.88 |
| AT2G14890 | arabinogalactan-protein (AGP9) | 266588_at | 0.14 | 1.09 |
| AT2G20530 /// AT4G28510 | prohibitin, putative | 263375_s_at | 0.16 | 0.68 |
| AT2G25530 | AFG1-like ATPase family protein | 265634_at | 11.68 | 0.78 |
| AT2G28000 | RuBisCO subunit binding-protein alpha subunit, chloroplast / 60 kDa chaperonin alpha subunit / CPN-60 alpha | 264069_at | 0.18 | 0.8 |
| AT2G29340 | short-chain dehydrogenase/reductase (SDR) family protein | 266265_at | 6.77 | 0.95 |
| AT2G35370 | glycine cleavage system H protein 1, mitochondrial (GDCSH) (GCDH) | 266636_at | 0.19 | 0.62 |
| AT2G39470 | photosystem II reaction center PsbP family protein | 266979_at | 0.15 | 0.92 |
| AT3G05910 | pectinacetylesterase, putative | 258750_at | 0.17 | 0.69 |
| AT3G07700 | ABC1 family protein | 259226_at | 7.66 | 0.92 |
| AT3G10420 | sporulation protein-related | 258925_at | 11.49 | 1.61 |
| AT3G14240 | subtilase family protein | 258368_at | 0.19 | 1.05 |
| AT3G17820 | glutamine synthetase (GS1) | 258160_at | 0.2 | 0.77 |
| AT3G18680 | aspartate/glutamate/uridylate kinase family protein | 257756_at | 0.09 | 0.91 |
| AT3G24190 | ABC1 family protein | 257253_at | 6.29 | 0.98 |
| AT3G25690 | hydroxyproline-rich glycoprotein family protein | 256754_at | 6.21 | 1.04 |
| AT3G30180 | cytochrome P450, putative | 256598_at | 6.11 | 0.94 |
| AT3G45010 | serine carboxypeptidase III, putative | 252606_at | 13.37 | 1.1 |
| AT3G47650 | bundle-sheath defective protein 2 family / bsd2 family | 252409_at | 0.16 | 1.15 |
| AT3G53130 /// AT3G53140 | cytochrome P450 family protein | 251979_at | 0.1 | 0.81 |
| AT3G53300 | cytochrome P450 family protein | 251988_at | 67.54 | 0.5 |
| AT4G02060 | prolifera protein (PRL) / DNA replication licensing factor Mcm7 (MCM7) | 255513_at | 0.2 | 0.78 |
| AT4G04590 | transposable element gene | 255313_at | 7.5 | 3.22 |
| AT4G09030 | arabinogalactan-protein (AGP10) | 255080_at | 0.14 | 0.36 |
| AT4G09760 | choline kinase, putative | 254998_at | 8.17 | 0.43 |
| AT4G14890 | ferredoxin family protein | 245347_at | 0.17 | 0.74 |
| AT4G19170 | 9-cis-epoxycarotenoid dioxygenase, putative / neoxanthin cleavage enzyme, putative / carotenoid cleavage dioxygenase, putative | 254564_at | 5.13 | 0.36 |
| AT4G21590 | bifunctional nuclease, putative | 254391_at | 0.2 | 2.35 |
| AT4G22010 | multi-copper oxidase type I family protein | 254363_at | 0.07 | 1.18 |
| AT4G25300 /// AT4G25310 | oxidoreductase, 2OG-Fe(II) oxygenase family protein | 254053_s_at | 10.62 | 1.56 |
| AT4G26780 | co-chaperone grpE family protein | 253949_at | 0.16 | 0.78 |
| AT4G33700 | CBS domain-containing protein | 253351_at | 5.25 | 0.8 |
| AT4G35060 | heavy-metal-associated domain-containing protein / copper chaperone (CCH)-related | 253172_at | 22.02 | 1.15 |
| AT4G35850 | pentatricopeptide (PPR) repeat-containing protein | 253116_at | 0.15 | 0.74 |
| AT4G38370 | phosphoglycerate/bisphosphoglycerate mutase family protein | 252995_at | 0.19 | 0.85 |
| AT5G03300 | adenosine kinase 2 (ADK2) | 250898_at | 0.17 | 0.54 |
| AT5G04900 | short-chain dehydrogenase/reductase (SDR) family protein | 250812_at | 0.19 | 0.84 |
| AT5G05600 | oxidoreductase, 2OG-Fe(II) oxygenase family protein | 250793_at | 0.13 | 0.29 |
| AT5G07010 | sulfotransferase family protein | 250662_at | 0.1 | 0.67 |
| AT5G09650 | inorganic pyrophosphatase family protein | 250496_at | 0.07 | 0.51 |
| AT5G11650 | hydrolase, alpha/beta fold family protein | 250335_at | 7.73 | 0.81 |
| AT5G13520 | peptidase M1 family protein | 245849_at | 0.16 | 0.82 |
| AT5G22630 | prephenate dehydratase family protein | 249910_at | 8.87 | 1.1 |
| AT5G22640 | MORN (Membrane Occupation and Recognition Nexus) repeat-containing protein | 249900_at | 0.15 | 0.78 |
| AT5G23210 | serine carboxypeptidase S10 family protein | 249847_at | 0.09 | 0.44 |
| AT5G24160 | squalene monooxygenase 1,2 / squalene epoxidase 1,2 (SQP1,2) | 249775_at | 6.49 | 1.3 |
| AT5G24580 | copper-binding family protein | 249755_at | 8.35 | 1.29 |
| AT5G37580 | tropomyosin-related | 249628_at | 0.17 | 0.77 |
| AT5G40260 | nodulin MtN3 family protein | 249401_at | 0.17 | 1.57 |
| AT5G41080 | glycerophosphoryl diester phosphodiesterase family protein | 249337_at | 0.19 | 1.5 |
| AT5G41780 | myosin heavy chain-related | 249270_at | 5.67 | 0.8 |
| AT5G42230 | serine carboxypeptidase S10 family protein | 249241_at | 75.37 | 1.53 |
| AT5G43580 | protease inhibitor, putative | 249101_at | 14 | 0.58 |
| AT5G45040 | cytochrome c6 (ATC6) | 248975_at | 0.13 | 1.09 |
| AT5G45650 | subtilase family protein | 248961_at | 0.07 | 1.15 |
| AT5G45940 | MutT/nudix family protein | 248923_at | 6.51 | 1.4 |
| AT5G49555 | amine oxidase-related | 248613_at | 0.18 | 0.66 |
| AT5G51260 | acid phosphatase, putative | 248440_at | 5.02 | 0.81 |
| AT5G52330 | meprin and TRAF homology domain-containing protein / MATH domain-containing protein | 248354_at | 6.71 | 1.1 |
| AT5G52970 | thylakoid lumen 15.0 kDa protein | 248287_at | 0.19 | 1.05 |
| AT5G63310 | nucleotide diphosphate kinase II, chloroplast (NDPK2) | 247376_at | 0.12 | 0.72 |
| AT5G66170 | senescence-associated family protein | 247136_at | 0.16 | 0.65 |
|  |  |  |  |  |
| *Uncharacterized/Hypothetical Proteins* | |  | MLN^a^  ILN | MLN  MMN |
| AT1G08800 | expressed protein | 264800_at | 5.7 | 0.76 |
| AT1G10650 | expressed protein | 261832_at | 6.67 | 1.13 |
| AT1G11170 | expressed protein | 262478_at | 8.95 | 1.12 |
| AT1G12080 | expressed protein | 264342_at | 0.11 | 0.86 |
| AT1G14620 | expressed protein | 260781_at | 0.09 | 0.63 |
| AT1G15400 | expressed protein | 262592_at | 5.46 | 2.05 |
| AT1G16000 | expressed protein | 261790_at | 0.17 | 0.69 |
| AT1G16720 | expressed protein | 255764_at | 8.84 | 1.36 |
| AT1G20070 | expressed protein | 261247_at | 42.73 | 0.26 |
| AT1G24600 | expressed protein | 265024_at | 12.26 | 0.81 |
| AT1G35170 /// AT1G35180 | expressed protein | 245783_s_at | 5.77 | 1.02 |
| AT1G48330 | expressed protein | 262236_at | 0.08 | 0.7 |
| AT1G56320 | expressed protein | 256217_at | 7.16 | 2.06 |
| AT1G67330 | expressed protein | 264998_at | 8.47 | 0.86 |
| AT1G68440 | expressed protein | 259856_at | 6.23 | 1.88 |
| AT1G79270 | expressed protein | 264102_at | 15.86 | 1.61 |
| AT2G19850 /// AT3G54730 | hypothetical protein | 266680_s_at | 5.74 | 0.5 |
| AT2G20585 | expressed protein | 263712_at | 0.2 | 0.7 |
| AT2G21180 | expressed protein | 264024_at | 13.03 | 2.37 |
| AT2G21185 | expressed protein | 264022_at | 7.7 | 2.3 |
| AT2G21640 | expressed protein | 263515_at | 0.14 | 1.12 |
| AT2G32180 /// AT2G32650 | expressed protein | 267553_s_at | 0.15 | 0.67 |
| AT2G33180 | expressed protein | 245165_at | 0.2 | 0.95 |
| AT2G33520 | expressed protein | 255840_at | 11.19 | 2.62 |
| AT2G34600 | expressed protein | 266901_at | 0.16 | 0.65 |
| AT2G34640 | expressed protein | 266957_at | 0.2 | 1.03 |
| AT2G43060 | expressed protein | 265245_at | 12.17 | 1.13 |
| AT3G02500 | expressed protein | 258503_at | 0.2 | 1.29 |
| AT3G09470 | expressed protein | 258699_at | 0.18 | 0.86 |
| AT3G10320 | expressed protein | 259150_at | 18.17 | 1.68 |
| AT3G17160 | expressed protein | 257879_at | 0.17 | 1.34 |
| AT3G22210 | expressed protein | 256796_at | 0.19 | 0.88 |
| AT3G22540 | expressed protein | 256926_at | 7.93 | 0.99 |
| AT3G23170 | expressed protein | 257925_at | 7.9 | 1.11 |
| AT3G25640 | expressed protein | 256759_at | 10.22 | 0.58 |
| AT3G48500 | expressed protein | 252362_at | 0.14 | 0.8 |
| AT3G48690 | expressed protein | 252315_at | 28.25 | 1.12 |
| AT3G57950 | expressed protein | 251612_at | 14.86 | 3.93 |
| AT3G59900 | expressed protein | 251436_at | 8.97 | 0.63 |
| AT3G60780 | expressed protein | 251339_at | 17.44 | 1.3 |
| AT4G00950 | expressed protein | 255652_at | 6.84 | 1.28 |
| AT4G11100 | expressed protein | 254911_at | 0.15 | 0.8 |
| AT4G17840 | expressed protein | 254691_at | 5.54 | 1.33 |
| AT4G18425 | expressed protein | 254629_at | 9.37 | 0.93 |
| AT4G24700 | expressed protein | 254145_at | 5.39 | 3.12 |
| AT4G27030 | expressed protein | 253943_at | 9.61 | 0.76 |
| AT4G27657 | expressed protein | 253859_at | 14.35 | 1.52 |
| AT4G27660 | expressed protein | 253883_at | 7.07 | 1.05 |
| AT4G30850 | expressed protein | 253590_at | 0.18 | 0.86 |
| AT4G33560 | expressed protein | 253298_at | 8.06 | 1.25 |
| AT5G02580 | expressed protein | 251012_at | 7.51 | 2.82 |
| AT5G04470 | expressed protein | 250844_at | 0.08 | 0.64 |
| AT5G13720 | expressed protein | 250247_at | 6.29 | 1.19 |
| AT5G16060 | expressed protein | 246479_at | 0.19 | 0.56 |
| AT5G24860 | expressed protein | 246967_at | 5.46 | 0.51 |
| AT5G24990 /// AT5G25020 | expressed protein | 246979_s_at | 5.05 | 1.44 |
| AT5G43150 | expressed protein | 249134_at | 13.96 | 0.93 |
| AT5G43180 | expressed protein | 249136_at | 11.59 | 1.01 |
| AT5G47050 | expressed protein | 248819_at | 7.83 | 1.85 |
| AT5G50100 | expressed protein | 248537_at | 5.51 | 1.13 |
| AT5G54970 | expressed protein | 248139_at | 0.17 | 0.87 |
| AT5G59510 | expressed protein | 247704_at | 6.96 | 1.6 |
| AT5G61340 | expressed protein | 247522_at | 7.36 | 1.16 |
| AT5G67370 | expressed protein | 246998_at | 12.43 | 1.88 |
| ^a^ All genes displaying a 5-fold or greater difference in probe signal value between MLN and ILN are shown (t test p-value cutoff 0.05, and FDR q-value cutoff 0.05). The probe signal ratios between MLN and MMN are shown for reference in the right hand column. | | | | |
