# Supplementary material for: Uncovering the Arabidopsis thaliana nectary transcriptome: investigation of differential gene expression in floral nectariferous tissues
Source: BMC Plant Biol. 2009 Jul 15;9:92. doi: 10.1186/1471-2229-9-92 (PMC2720969; doi:10.1186/1471-2229-9-92)
Supplement: Additional file 10 — Genes displaying differential expression between mature median and mature lateral nectaries. All genes displaying a 5-fold difference in probe signal value between MMN and MLN are shown (t test p-value cutoff 0.05, and FDR q-value cutoff 0.05). [file 1471-2229-9-92-S10.doc]

| **Additional file 10 - Genes displaying differential expression between mature median and mature lateral nectaries** | | | | | |
| --- | --- | --- | --- | --- | --- |
| Locus | TAIR Annotation | Probe Set | MMN^a^  MLN | ILN  MLN | |
| *Transcription Factors* | |  |  |  | |
| AT1G25330 | basic helix-loop-helix (bHLH) family protein | 245640_at | 13.08 | 0.78 | |
| AT2G16720 | myb family transcription factor | 265359_at | 0.19 | 0.17 | |
| AT2G46870 | DNA-binding protein, putative | 266760_at | 18.3 | 0.85 | |
| AT4G28140 | AP2 domain-containing transcription factor, putative | 253799_at | 8.37 | 0.36 | |
|  |  |  |  |  | |
| *Carbohydrate Metabolism* | |  |  |  | |
| AT3G17130 | invertase/pectin methylesterase inhibitor family protein | 257876_at | 15.74 | 0.39 | |
|  |  |  |  |  | |
| *Signaling and Development* | | |  | |  |
| AT1G76040 | calcium-dependent protein kinase, putative / CDPK, putative | 262671_at | 7.95 | 1.02 | |
|  |  |  |  |  | |
| *Hormone Metabolism and Response* | |  |  |  | |
| AT4G25420 | gibberellin 20-oxidase | 254065_at | 8.86 | 0.39 | |
| AT5G15230 | gibberellin-regulated protein 4 (GASA4) / gibberellin-responsive protein 4 | 250109_at | 8.19 | 3.57 | |
|  |  |  |  |  | |
| *Transporter and Channel Proteins* | |  |  |  | |
| AT1G73220 | sugar transporter family protein | 260097_at | 41.66 | 1.98 | |
| AT2G37170 /// AT2G37180 | plasma membrane intrinsic protein 2B (PIP2B) / aquaporin PIP2.2 (PIP2.2) | 265444_s_at | 6.08 | 0.29 | |
| AT5G14940 | Proton-dependent oligopeptide transport (POT) family protein | 246566_at | 8.5 | 0.16 | |
|  |  |  |  |  | |
| *Response to Biotic and Abiotic Factors, Defense* | |  |  |  | |
| AT1G52690 | late embryogenesis abundant protein, putative / LEA protein, putative | 262128_at | 5.90 | 0.05 | |
| AT2G19970 | pathogenesis-related protein, putative | 265588_at | 5.38 | 2.20 | |
| AT3G52470 | Harpin-induced family protein / HIN1 family protein / harpin-responsive family protein | 252058_at | 5.03 | 1.30 | |
|  |  |  |  |  | |
| *Other* | |  |  |  | |
| AT1G20160 | subtilase family protein | 261224_at | 6.62 | 0.96 | |
| AT1G21460 | nodulin MtN3 family protein | 260876_at | 6.15 | 0.88 | |
| AT1G65450 | transferase family protein | 264160_at | 5.94 | 1 | |
| AT1G74820 | cupin family protein | 262214_at | 65.44 | 0.4 | |
| AT2G02000 /// AT2G02010 | glutamate decarboxylase, putative | 265221_s_at | 19.9 | 0.75 | |
| AT2G19900 | Malate oxidoreductase, putative | 266690_at | 5.45 | 0.04 | |
| AT3G55290 /// AT3G55310 | short-chain dehydrogenase/reductase (SDR) family protein | 251780_s_at | 7.64 | 1.32 | |
| AT4G33110 /// AT4G33120 | coclaurine N-methyltransferase, putative | 253362_s_at | 5.15 | 2.26 | |
| AT5G17330 | glutamate decarboxylase 1 (GAD 1) | 250090_at | 7.53 | 0.53 | |
|  |  |  |  |  | |
| *Uncharacterized/Hypothetical Proteins* | |  |  |  | |
| AT1G13930 | expressed protein | 262609_at | 14.00 | 0.74 | |
| AT5G64510 | expressed protein | 247293_at | 5.97 | 0.68 | |
|  |  |  |  |  | |
| ^a^ All genes displaying a 5-fold difference in probe signal value between MMN and MLN are shown (t test p-value cutoff 0.05, and FDR q-value cutoff 0.05).. The probe signal ratios between ILN and MLN are shown for reference in the right hand column. | | | | | |
