# Supplementary figures and images for: Uncovering the Arabidopsis thaliana nectary transcriptome: investigation of differential gene expression in floral nectariferous tissues
Source: BMC Plant Biol. 2009 Jul 15;9:92. doi: 10.1186/1471-2229-9-92 (PMC2720969; doi:10.1186/1471-2229-9-92)

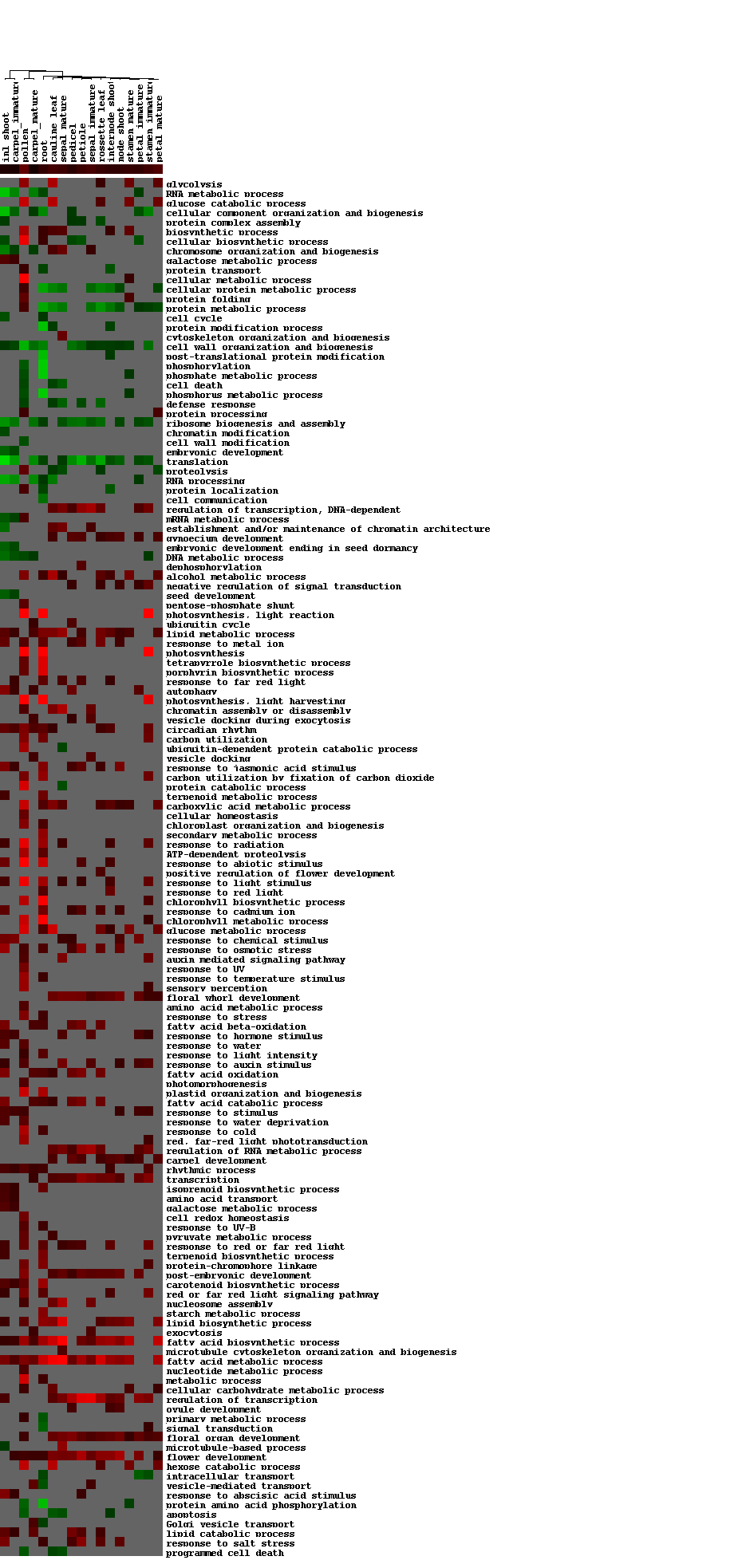

Supplement: Additional file 12 — Full gene ontology heat map. Full heat map of gene ontology analysis for nectary-enriched genes. [file 1471-2229-9-92-S12.bmp]
